# Association of Lipoprotein(a) With Cardiovascular and Cerebrovascular Disease in a Nationally Representative Cohort of Germany

**DOI:** 10.1016/j.jacadv.2025.102015

**Published:** 2025-07-23

**Authors:** Gloria G. Gelfert, Ulrike Grittner, Ronny Kuhnert, Christa Scheidt-Nave, Matthias Endres, Alexander H. Nave

**Affiliations:** a Center for Stroke Research Berlin (CSB), Charité– Universitätsmedizin Berlin, Corporate Member of Freie Universität Berlin and Humboldt Universität zu Berlin, Berlin, Germany; b Department of Neurology with Experimental Neurology, Charité– Universitätsmedizin Berlin, Corporate Member of Freie Universität Berlin and Humboldt Universität zu Berlin, Berlin, Germany; c Institute of Biometry and Clinical Epidemiology, Charité Universitätsmedizin Berlin, Berlin, Germany; d Berlin Institute of Health (BIH) at Charité, Charité– Universitätsmedizin Berlin, Corporate Member of Freie Universität Berlin and Humboldt Universität zu Berlin, Berlin, Germany; e Department of Epidemiology and Health Monitoring, Robert Koch Institute, Berlin, Germany; f German Centre for Cardiovascular Research (DZHK), Partner Site Berlin, Berlin, Germany; g German Center for Neurodegenerative Diseases (DZNE), Partner Site Berlin, Berlin, Germany; h German Center for Mental Health (DZPG), Partner Site Berlin, Berlin, Germany

**Keywords:** cardiovascular disease, cardiovascular risk, epidemiology, lipoprotein(a), prevention, venous thrombosis

## Abstract

**Background:**

Population-based data on the distribution of lipoprotein(a) (Lp[a]) within the German population are lacking.

**Objectives:**

The aim of the study was to determine the age- and sex-specific Lp(a) distribution in Germany and analyze its association with different types of cardiovascular disease (CVD).

**Methods:**

We analyzed cross-sectional data from the German National Health Interview and Examination Survey 1998, a population-based study representing the German adult population’s health status from 1997 to 1999. We examined serum Lp(a) according to demographics and investigated associations between Lp(a) and history of self-reported CVD. We tested Lp(a) in spline analysis on continuous scales and as dichotomous Lp(a) thresholds.

**Results:**

In the German National Health Interview and Examination Survey 1998 (n = 6,657), median Lp(a) was 15.3 mg/dL (Q1-Q3: 5.6, 43.1). Lp(a) levels ≥50 mg/dL were present in 21.6%. Men had substantially higher median Lp(a) than women (22.1 mg/dL vs 10.3 mg/dL). Median Lp(a) levels were significantly higher in individuals with than without a history of atherosclerotic cardiovascular disease (ASCVD) but did not significantly differ between people with and without venous thrombosis. In logistic regression analysis with splines, higher Lp(a) levels were associated with a higher probability of a history of CVD. In logistic regression analyses, Lp(a) ≥50 mg/dL was associated with a history of ASCVD (OR: 1.36 [95% CI: 1.04-1.78]; *P* = 0.023).

**Conclusions:**

About 1 in 5 German individuals from a prestatin era population had Lp(a) levels ≥50 mg/dL. Higher levels were independently associated with ASCVD, but not with venous thrombosis. These findings provide a basis for future prospective studies to define the role of Lp(a) in CVD risk in the German population.

Elevated lipoprotein(a) (Lp[a]) is considered to be a causal risk factor for atherosclerotic cardiovascular disease (ASCVD), including coronary artery disease and ischemic stroke.^1^ The evidence regarding the association between Lp(a) and venous thrombosis (VT) has been conflicting. Lp(a) is a lipoprotein composed of a low-density lipoprotein-like particle covalently linked to apolipoprotein(a) (apo[a]), a plasminogen-like glycoprotein.^1^ Lp(a) levels are primarily determined by the *LPA* gene, which encodes apo(a). Lp(a) can be found in multiple isoforms, and there is an inverse relationship between apo(a) isoform size and Lp(a) serum concentration. The high heterogeneity of the apo(a) size among individuals results from a polymorphism of the kringle IV-type 2 repeat within the apo(a) protein.^2^ Lp(a) has an approximately 6-fold higher pro-atherogenic potential than low-density lipoprotein, as it transports a greater number of oxidized phospholipids, by which it contributes proinflammatory, prothrombotic, and procalcifying signals to cells.^1^ While Lp(a) is not modifiable by lifestyle changes, levels can even increase through statin therapy.^3^

Multiple observational studies and meta-analyses have confirmed Lp(a) as an independent risk factor for cardiovascular and cerebrovascular disease using various cutoff levels and continuous scales.^4^, ^5^, ^6^ In first-time stroke patients, for example, Lp(a) levels >30 mg/dL or >100 nmol/L were associated with an increased recurrent vascular risk, indicating that elevated Lp(a) represents an important risk factor for residual vascular risk after ischemic stroke.^7^^,^^8^ Data from the UK Biobank highlighted a linear association of Lp(a) and incident ASCVD risk, demonstrating that Lp(a) levels >150 nmol/L increase the risk of major adverse cardiovascular events, especially nonfatal myocardial infarction (MI) and coronary revascularization in patients with prior ASCVD.^9^^,^^10^

Although studies have shown a largely continuous association between Lp(a) and the risk for ASCVD, current international guidelines recommend a clinically relevant threshold for elevated Lp(a) as ≥50 mg/dL (125 nmol/L).^5^^,^^11^ Mendelian randomization studies have provided additional evidence for a causal relationship between genetically predicted high levels of Lp(a) and increased risk for ASCVD.^12^^,^^13^ Patients with extremely high Lp(a) levels >180 mg/dL (>430 nmol/L) have a lifetime risk of ASCVD equivalent to the risk associated with heterozygous familial hypercholesterolemia.^14^ The guidelines therefore recommend that everyone should have their Lp(a) level checked once in their lifetime. However, screening to identify people at risk remains inadequately implemented by most physicians. An analysis of health insurance claims in Germany revealed that Lp(a) testing was merely performed in <0.5% of the general population and <1% of patients with established ASCVD.^15^ However, a recent study at Johns Hopkins Hospital showed a steady increase in Lp(a) testing over time, especially in populations at risk.^16^

The distribution of Lp(a) across different populations has previously been studied: a recent epidemiological study from the U.S. population has found that 14.7% had Lp(a) levels ≥50 mg/dL.^17^ In contrast, the Copenhagen General Population Study found 20.0% of adults had Lp(a) levels ≥50 mg/dL.^18^ The population-based SHIP-0 cohort from northeast Germany had a median Lp(a) of 9.4 mg/dL, whereas a large study from a cardiovascular referral center in Germany found that 18.4% of 52,898 patients with CVD had levels >50 mg/dL.^19^^,^^20^ Thus, Lp(a) is regarded as the most prevalent monogenetic risk factor for ASCVD worldwide. Still, epidemiological data on Lp(a) in the German general population, its associations with different CVDs in a large population-based cohort, and age- and sex-specific Lp(a) thresholds for risk stratification are lacking. The aim of this study was to explore the age- and sex-specific distribution of Lp(a) in the German population by using available data from a nationally representative cohort conducted in the prestatin era (before 2000s). We further investigate the association between the history of different types of CVD as well as VT with different Lp(a) levels (Central Illustration).

**Central Illustration:**
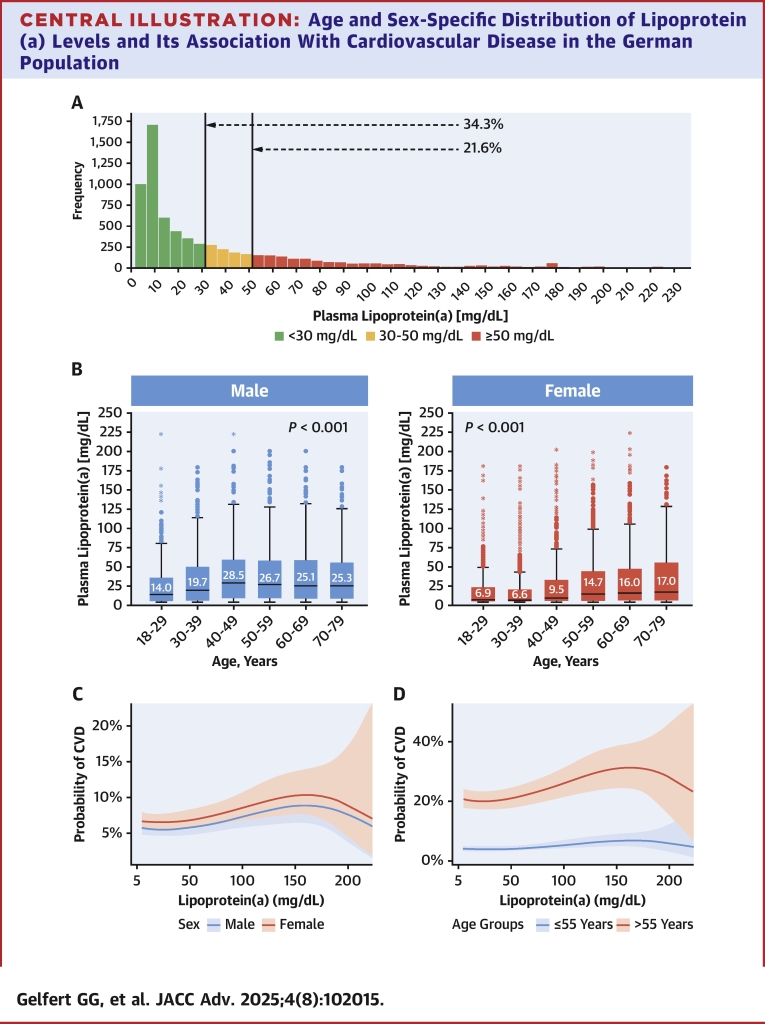
Age and Sex-Specific Distribution of Lipoprotein(a) Levels and Its Association With Cardiovascular Disease in the German Population All analyses were based on 6,657 individuals (aged 18-79 years) from the German National Health Interview and Examination Survey 1998 (GNHIES98). (A) Distribution of Lp(a) among adults in Germany. (B) Median Lp(a) in Germany across sex and age categories. Women: n = 3,443; men: n = 3,214. Bars indicate median and IQR. *P* values are calculated with the Kruskal-Wallis test. (C) Spline analysis of the association between history of CVD and Lp(a) concentration by sex (n = 6,434; 3,302 women; 3,132 men). (D) Spline analysis of the association between history of CVD and Lp(a) concentration by age groups (n = 6,434; ≤55 years, 4,443; >55 years, 1,991). Abbreviations as in Figure 3.

## Methods

### Study design and population

An observational, retrospective, cross-sectional analysis was performed using data from the German National Health Interview and Examination Survey 1998 (GNHIES98), a representative study of the German adult population’s health status. The study was carried out by the Robert Koch Institute between October 1997 and March 1999 at 120 study sites across Germany. A population-based sample of adults aged 18 to 79 years was drawn from local population registries. Overall, 7,124 persons (3,450 men and 3,674 women) participated. The survey included written health questionnaires, personal medical interviews, physical examinations, and laboratory analyses of blood samples. The study was based on a two-stage stratified cluster sampling. The data were weighted, adjusting for design-related selection probabilities and the varying participation behavior in different population groups in Germany. The study concept and design have been previously described.^21^, ^22^, ^23^

All GNHIES98 participants with available Lp(a) testing were eligible for the present analysis (n = 6,657). The implementation of the survey conforms to the principles of the Helsinki Declaration and was consented to by the Federal Commissioner for Data Protection, Germany. All participants provided written informed consent prior to the interview and examination. This study was approved by the ethics committee of the Charité-Universitätsmedizin Berlin (EA4/112/22).

### Study variables and definitions

Elevated Lp(a) levels were defined at the cutoff value of 50 mg/dL (low Lp[a]: <50 mg/dL, high Lp[a]: ≥50 mg/dL), as this is the recognized value in current guidelines for increased cardiovascular risk.^11^ In GNHIES98, medical history was obtained and recorded during a standardized computer-assisted personal interview (CAPI) conducted face-to-face by a physician.

We used medical history of nonfatal MI, coronary heart disease, heart failure, stroke, circulatory disorder of the brain, circulatory disorder of the legs, and VT to define cardiovascular health outcomes. Information was summarized and categorized into the following groups: any cardiovascular disease (CVD) = history of one or more cardiovascular health outcomes; ASCVD = history of CVD without consideration of VT; heart disease (history of MI, coronary heart disease, or heart failure); and cerebrovascular disease (history of stroke or cerebral circulatory disorder). The type and cause of stroke were not recorded. Circulatory disorders of the brain were defined as events that were accompanied by paralysis, sensory disorders/paresthesia, or speech disorders and were not due to migraine. Circulatory disorder of the legs was defined as peripheral artery disease. Arterial hypertension was defined as systolic blood pressure ≥140 mm Hg or diastolic blood pressure ≥90 mm Hg (measured during the physical examination) or use of antihypertensive medication in the past 7 days with prior physician-diagnosed arterial hypertension (as surveyed in the CAPI). Diabetes mellitus was defined as physician-diagnosed diabetes mellitus (type 1 and 2, as surveyed in the CAPI) or the use of antidiabetic medication (oral glucose-lowering drugs or insulin) in the past 7 days preceding the examination. Dyslipidemia was defined as physician-diagnosed elevated blood lipid or cholesterol levels (as surveyed in the CAPI). Lipid-lowering medication included statins (HMG-CoA reductase inhibitors), fibrates, nicotinic acid, bile acid sequestrants, and other agents affecting lipid metabolism (such as omega-3 fatty acids or essential phospholipids). All drugs with the Anatomical Therapeutic Chemical classification system code C10 were identified as lipid-lowering medications. PCSK9 inhibitors were not yet developed at the time of the study and therefore were not included.

### Lp(a) measurement

Serum samples from patients were retrieved at each study site. The obtained blood samples were processed within 1 hour after retrieval and stored at −40 °C until the analysis was performed at the central laboratory at the Robert Koch Institute, Berlin. Serum lipid analyses, including Lp(a) measurements, were performed consecutively within several weeks upon retrieval. Lp(a) was quantified using an immunoturbidimetric assay method with Cobas Mira clinical chemistry analyzer (Roche Diagnostics Systems). Lp(a) serum concentration was measured in mg/L and is reported as mg/dL.

### Statistical analysis

Continuous variables were generally presented as mean (SD). As Lp(a) showed considerable deviation from normal distribution, summary measures are presented as median (IQR). Summary measures for nominal variables are presented as absolute and relative frequencies. We compared median Lp(a) levels by sex and strata of age as well as health-related categorical baseline variables. For unadjusted association analyses, the Student’s *t*-test and the Mann-Whitney *U* test or Kruskal-Wallis test were used depending on the distribution of the data. To analyze the association of elevated Lp(a) with categorical baseline parameters, Pearson chi-square tests were used to compare group differences in prevalence estimates of elevated serum Lp(a) levels in different subgroups.

We used piecewise polynomial splines with 3 degrees of freedom and without implementation of boundary constraints to explore the potentially nonlinear association of Lp(a) and the history of CVD. These spline analyses were performed using binary logistic regression models of cardiovascular health outcomes as dichotomous outcome variables (yes/no) adjusting for age, sex, and cardiovascular risk factors (as dichotomous variables) including arterial hypertension, dyslipidemia, diabetes mellitus, overweight (body mass index >25 kg/m^2^), smoking (ever), and lipid-lowering medication. Furthermore, we evaluated for statistical interaction by modeling the interaction terms “Lp(a) × sex”, “Lp(a) x age” and “Lp(a) x diabetes” and compared model performances with and without interaction terms and splines using the Akaike information criterion. Final models were chosen according to the smallest Akaike information criterion. Before conducting the piecewise polynomial spline analysis to assess the association between Lp(a) and the history of CVD, we carefully considered the standard assumptions for this method. We visually inspected the fitted spline curves to confirm smoothness. We furthermore investigated associations between Lp(a) and CVDs using multivariable logistic regression analyses, where associations were tested in dichotomous Lp(a) categories (≥30 mg/dL, ≥50 mg/dL, ≥70 mg/dL, and ≥100 mg/dL) in a sensitivity analysis. The cutoffs of 150 mg/dL and 180 mg/dL were not included in this analysis due to insufficient group sizes. As the distribution of Lp(a) values was right-skewed, it was log-transformed before regression analysis. Exploratory subgroup analyses were conducted for clinically pertinent populations, contingent upon sufficient group sizes. Groups were stratified by age (>55 vs ≤55 years) or sex, with interaction testing performed when group sizes permitted.

As a sensitivity analysis, propensity score matching was done for each of the event histories separately to account for possible confounding. The propensity scores for the matching were based on binary logistic regressions to estimate the probability of having a history of an event with the covariates age, sex, body mass index category, smoking status, arterial hypertension, diabetes mellitus, dyslipidemia, and use of lipid-lowering medication. Participants were matched exactly for sex and dyslipidemia, followed by 1:5 nearest neighbor matching (0.2 SD of log-odds caliper) without replacement. After matching, we analyzed Lp(a) level differences between individuals with and without a history of an event using linear mixed models. The models used log-transformed Lp(a) as the dependent variable, with age, sex, their interaction, and event status (yes/no) as independent variables. Random intercepts for matching groups were also included.

This is an exploratory study. No adjustment for multiple testing was implemented. Interpretation of results was primarily based on effect estimates and 95% CIs. All results were weighted using sampling weights in order to obtain results representative of the general population in Germany. Unique sampling weights were computed for each individual participating in the GNHIES98 based on population statistics 31.12.1997 as the reciprocal of sampling probability within subgroups of sex, 5-year age group, community size, and federal state.^17^ Where possible, survey procedures were used to account for complex sample design. For data analysis, we used IBM SPSS Statistics 29 (IBM Corporation) and RStudio (version 2023.12.1 + 402, Posit Software, PBC).^24^, ^25^, ^26^, ^27^

## Results

### Distribution of Lp(a) in the study population

**Figure 1:**
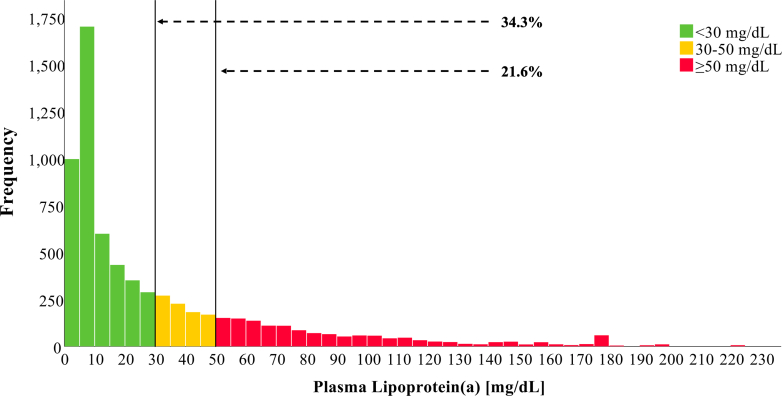
**Distribution of Lipoprotein(a) Among Adults in Germany** Analyses were based on 6,657 individuals (aged 18-79 years) from the German National Health Interview and Examination Survey 1998. Arrows indicate that 34.3% had Lp(a) levels ≥30 mg/dL, and 21.6% had Lp(a) levels ≥50 mg/dL. Lp(a) = lipoprotein(a).

Among the GNHIES98 population (n = 7,124), valid Lp(a) data were available for 6,657 participants. The mean age of the study population was 45 years, and 50.8% were female. Table 1 displays pertinent baseline characteristics of the study population. Of the GNHIES98 participants, 13.2% had a history of CVD and 4.7% had lipid-lowering therapy. Lp(a) levels ranged from 4.5 to 222.5 mg/dL with a median Lp(a) of 15.3 mg/dL (Q1-Q3: 5.6, 43.1). Lp(a) levels ≥30 mg/dL were present in 34.3% of individuals, and 21.6% had levels ≥50 mg/dL (Figure 1). The 95th percentile of Lp(a) serum levels was at 111.8 mg/dL (n = 319).

**Table 1:** Baseline Characteristics of the Study Population GNHIES98 (N = 6,657) in Germany

		% Missing (n)
Age (y)	45.4 (16.1)	0.0% (0)
Female	50.8% (3,443)	0.0% (0)
Anthropometric measures		
Body mass index (kg/m^2^)	26.7 (4.7)	0.5% (30)
Waist-to-hip ratio	0.86 (0.09)	0.8% (54)
Blood test levels		
Glucose (mmol/L)	5.5 (1.6)	0.0% (0)
HbA1c (%)	5.6 (0.9)	0.0% (76)
Lp(a) (mg/dL)∗	15.3 (5.6-43.1)	0.0% (0)
Lp(a) ≥30 mg/dL	34.3% (2,206)	0.0% (0)
Lp(a) ≥50 mg/dL	21.6% (1,385)	0.0% (0)
Lp(a) ≥70 mg/dL	13.4% (838)	0.0% (0)
Lp(a) ≥100 mg/dL	6.7% (427)	0.0% (0)
Lp(a) ≥150 mg/dL	2.2% (141)	0.0% (0)
Lp(a) ≥180 mg/dL	0.4% (20)	0.0% (0)
Total cholesterol (mmol/L)	6.0 (1.3)	0.0% (0)
LDL cholesterol (mmol/L)	3.8 (1.1)	3.1% (204)
HDL cholesterol (mmol/L)	1.5 (0.5)	0.0% (0)
Triglycerides (mmol/L)	1.7 (1.2)	0.0% (0)
Cardiovascular risk factors, % (n)		
Arterial hypertension	27.1% (1,890)	0.5% (35)
Diabetes mellitus	5.1% (340)	0.4% (24)
Dyslipidemia	22.6 (1,463)	0.4% (24)
BMI ≥25 kg/m^2^ (overweight)	60.6% (3,995)	0.5% (30)
BMI ≥30 kg/m^2^ (obesity)	21.2% (1,403)	0.5% (30)
Ever smoker	54.9% (3,543)	2.3% (156)
Alcohol consumption (g/d)∗	2.7 (0.4-13.1)	2.8% (189)
Alcohol consumption >20 g/d (men); >10 g/d (women)	19.1% (1,242)	2.8% (189)
History of CVDs, % (n)		
CVD	13.2% (869)	0.36% (24)
ASCVD	11.0% (712)	0.36% (24)
Heart failure	3.5% (209)	0.36% (24)
Heart disease	7.8% (508)	0.36% (24)
Myocardial infarction	2.2% (147)	0.36% (24)
Coronary heart disease	5.7% (382)	0.36% (24)
Cerebrovascular disease	2.4% (164)	0.36% (24)
Stroke	1.3% (89)	0.36% (24)
Cerebral circulatory disorder	1.6% (113)	0.36% (24)
Peripheral artery disease	3.0% (176)	0.36% (24)
Venous thrombosis	4.0% (263)	0.36% (24)
Medication, % (n)		
Lipid-lowering therapy	4.7% (306)	0.39% (26)
Antihypertensive medication	15.7% (1,051)	0.41% (27)
Glucose-lowering medication	3.2% (223)	0.41% (27)

Normally distributed continuous variables were presented as mean (SD) and categorical variables as % (n).

Results weighted for the German population structure as of December 31st, 1997, and adjusted for complex study design. n = unweighted and unadjusted sample size.

CVD: History of any cardiovascular disease, including self-reported myocardial infarction, coronary heart disease, stroke, cerebral circulatory disorder, peripheral artery disease, heart failure, or venous thrombosis.

ASCVD: History of any atherosclerotic cardiovascular disease, including self-reported myocardial infarction, coronary heart disease, heart failure, stroke, cerebral circulatory disorder, or peripheral artery disease.

ASCVD = atherosclerotic cardiovascular disease; BMI = body mass index; CVD = cardiovascular disease; GNHIES98 = German National Health Interview and Examination Survey 1998; HbA1c = hemoglobin A1c; HDL = high-density lipoprotein; Lp(a) = lipoprotein(a); LDL = low-density lipoprotein.

∗ Not normally distributed continuous variables were presented as median (IQR).

### Age- and sex-specific distribution of Lp(a)

**Figure 2:**
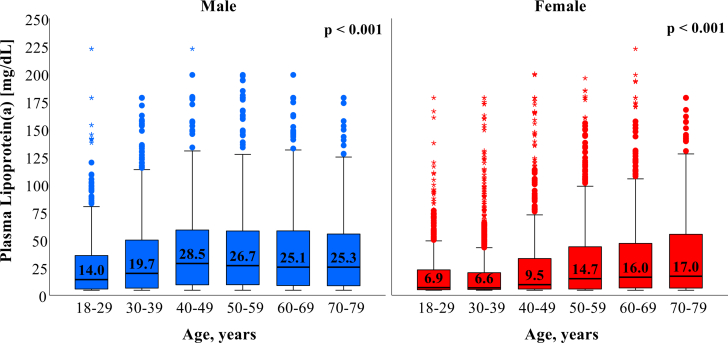
**Median Lipoprotein(a) in Germany Across Sex and Age Categories** Analyses were based on 6,657 individuals (aged 18-79 years) from the German National Health Interview and Examination Survey 1998. Women: n = 3,443; men: n = 3,214. Bars indicate median and IQR. *P* values are calculated with the Kruskal-Wallis test.

Median Lp(a) levels differed with regard to age, sex, and other clinical characteristics (Supplemental Table 1). In the total population, Lp(a) levels were higher in older age (Supplemental Figure 1). In the age group ≤55 years, median Lp(a) was 13.3 mg/dL compared to 20.5 mg/dL in individuals >55 years (*P* < 0.001). Across all age groups, median Lp(a) levels were distinctively higher in men than in women. Compared to younger individuals of the same sex, Lp(a) levels were higher in men after the age of 30 years, while in women, Lp(a) levels were predominantly higher after the age of 50 years (Figure 2).

Compared to women, men more frequently demonstrated Lp(a) levels ≥50 mg/dL (25.7% vs 17.6%), see Supplemental Table 2. Also, among participants without a history of CVD, men consistently had higher Lp(a) levels than women.

### Lp(a) distribution across different types of CVD

Median Lp(a) levels were generally higher in participants with cardiovascular risk factors and a history of CVD (Supplemental Tables 1 and 2). Participants with CVD were more likely to have Lp(a) levels ≥50 mg/dL compared to participants without CVD (28.8% vs 20.4%, *P* = 0.001) (Supplemental Table 2). Younger participants (≤55 years) with a history of ASCVD had substantially higher median Lp(a) levels (29.5 mg/dL) compared to participants without ASCVD (13.1 mg/dL) (Supplemental Figure 3B). Women with a history of CVD had almost twice as high Lp(a) levels compared to women without a history of CVD (Supplemental Figure 2A). In contrast to men, women with a history of VT also showed significantly higher Lp(a) levels compared to those without VT history (9.8 mg/dL vs 17.4 mg/dL), as depicted in Supplemental Figure 2C. For Lp(a) distribution of individual cardiovascular endpoints, please see the Supplemental Appendix.

### Cardiovascular disease-specific association with Lp(a)

**Figure 3:**
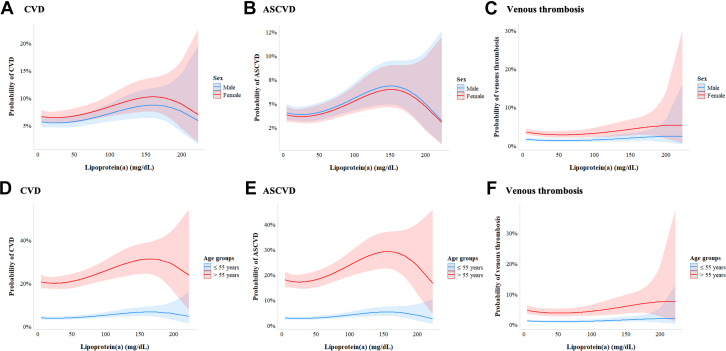
**Spline Analysis of the Association Between History of Cardiovascular Disease and Lipoprotein(a)** (A to C) Spline analysis of the association between history of CVD and Lp(a) concentration by sex in Germany (n = 6,434; 3,302 women; 3,132 men). (D to F) Spline analysis of the association between history of CVD and Lp(a) concentration by age groups in Germany (n = 6,434; ≤55 years, 4,443; >55 years, 1,991). Analyses are based on 6,435 individuals with complete data from the German National Health Interview and Examination Survey 1998. CVD: History of any cardiovascular disease, including self-reported myocardial infarction, coronary heart disease, stroke, cerebral circulatory disorder, peripheral artery disease, heart failure, or venous thrombosis. ASCVD: History of any atherosclerotic cardiovascular disease, including self-reported myocardial infarction, coronary heart disease, heart failure, stroke, cerebral circulatory disorder, or peripheral artery disease. Lp(a) = lipoprotein(a); ASCVD = atherosclerotic cardiovascular disease; CVD = cardiovascular disease.

Figure 3A shows the probability of CVD as a function of continuous Lp(a) stratified by sex, which demonstrates a steep increase in the presence of CVD at Lp(a) levels between 100 and 180 mg/dL. Figure 3D illustrates the same model stratified by age groups, demonstrating that older participants have a higher overall probability of CVD, which further increases with higher Lp(a) levels in this age group. The marginal ORs derived from a regression model with splines reveal a positive association of Lp(a) and the presence of CVD (Table 2). This becomes especially apparent at higher Lp(a) levels; for example, at a serum concentration of 100 mg/dL, the OR for the presence of CVD is 1.34 (95% CI: 1.05-1.70). Similarly, higher levels of Lp(a) were associated with the probability of existing ASCVD (Figure 3B), with an OR of 1.46 (95% CI: 1.13-1.89) at an Lp(a) serum concentration of 100 mg/dL. There was no clear association between Lp(a) and VT with an OR of 0.99 (95% CI: 0.68-1.46) (Figure 3C). Clear associations were observed between Lp(a) at 100 mg/dL and the probability of heart disease with an OR of 1.42 (95% CI: 1.07-1.88) and cerebrovascular disease with an OR of 1.35 (95% CI: 0.86-2.11). Adding an interaction term between sex and Lp(a), age and Lp(a), or Lp(a) and diabetes did not improve model performance. For spline analysis of all individual outcomes, see Supplemental Table 3 and Supplemental Figures 4 and 5.

**Table 2:** Spline Analysis of the Independent Association of Lp(a) Cut-Points With History of CVD, GNHIES98

Lp(a)	CVD (n = 857)		ASCVD (n = 674)		Venous Thrombosis (n = 252)	
	OR∗	95% CI	OR∗	95% CI	OR∗	95% CI
15 mg/dL	1.00		1.00		1.00	
30 mg/dL	1.01	0.92-1.10	1.00	0.91-1.10	0.91	0.80-1.05
50 mg/dL	1.05	0.92-1.21	1.07	0.92-1.25	0.87	0.70-1.09
70 mg/dL	1.14	0.97-1.35	1.19	0.99-1.43	0.89	0.68-1.16
100 mg/dL	1.33	1.05-1.69	1.45	1.13-1.87	0.99	0.68-1.46
150 mg/dL	1.68	1.21-2.32	1.86	1.32-2.63	1.33	0.80-2.23
180 mg/dL	1.76	1-16-2.67	1.83	1.17-2.85	1.55	0.84-2.88

Results weighted for the German population structure as of December 31st, 1997, and not adjusted for complex study design. n = unweighted, unadjusted sample size.

N = 6,435.

CVD: History of any cardiovascular disease, including self-reported myocardial infarction, coronary heart disease, stroke, cerebral circulatory disorder, peripheral artery disease, heart failure, or venous thrombosis.

ASCVD: History of any atherosclerotic cardiovascular disease, including self-reported myocardial infarction, coronary heart disease, stroke, cerebral circulatory disorder, or peripheral artery disease.

Abbreviations as in Table 1.

∗ OR: Marginal ORs from multivariable regression of Lp(a) with splines adjusted for sex, age, dyslipidemia, arterial hypertension, diabetes mellitus, BMI risk categories, smoking (ever), and lipid-lowering medication.

### Sensitivity analysis

Results of the sensitivity analysis, which tested Lp(a) as a dichotomous variable, are presented in Table 3. Elevated Lp(a) levels (≥50 mg/dL) were associated with a statistically nonsignificant increase in the odds of a history of CVD in the final multivariable logistic regression model (OR: 1.22 [95% CI: 0.97-1.55]; *P* = 0.089). In contrast, the presence of ASCVD showed a stronger and statistically significant association with elevated Lp(a) levels (OR: 1.36 [95% CI: 1.04-1.78]; *P* = 0.023), primarily driven by the exclusion of VT from this composite endpoint. No association of Lp(a) and a history of VT was observed. For all individual disease outcomes, see Supplemental Table 4.

**Table 3:** Independent Association of Lp(a) With History of CVD Applying 50 mg/dL as a Dichotomous Cut-Point, GNHIES98

	CVD (n = 857)		ASCVD (n = 674)		Venous Thrombosis (n = 252)	
	OR∗ (95% CI)	*P* Value	OR∗ (95% CI)	*P* Value	OR∗ (95% CI)	*P* Value
Lp(a) ≥50 mg/dL	1.26 (1.00-1.59)	0.049	1.40 (1.08-1.82)	0.011	0.87 (0.60-1.24)	0.425

Results weighted for the German population structure as of December 31st, 1997, and adjusted for complex study design. n = unweighted, unadjusted sample size.

N = 6,435.

CVD: History of any cardiovascular disease, including self-reported myocardial infarction, coronary heart disease, stroke, cerebral circulatory disorder, peripheral artery disease, heart failure, or venous thrombosis.

ASCVD: History of any atherosclerotic cardiovascular disease, including self-reported myocardial infarction, coronary heart disease, heart failure, stroke, cerebral circulatory disorder, or peripheral artery disease.

Abbreviations as in Table 1.

∗ ORs from multivariable logistic regression models adjusted for sex, age, dyslipidemia, arterial hypertension, diabetes mellitus, BMI risk categories, smoking (ever), and lipid-lowering medication.

In propensity score-matched regression analysis (n = 2,995), individuals with a history of ASCVD had higher marginal mean Lp(a) levels compared to those without (35.7 mg/dL [95% CI: 32.7-38.9] vs 31.6 mg/dL [95% CI: 29.9-33.4]; *P* = 0.014), and similar differences were found for those with a history of CVD compared to those without a history of CVD (34.0 mg/dL [95% CI: 31.4-36.8] vs 30.5 mg/dL [95% CI: 28.9-32.1]; *P* = 0.015). Comparing individuals with and without VT, similar Lp(a) levels were identified after propensity score matching (Supplemental Table 5). For detailed results, see Supplemental Tables 5 and 6 and Supplemental Figure 6.

## Discussion

This study is the first to evaluate the epidemiology of Lp(a) in a nationally representative cohort of Germany and to examine sex- and age-specific associations of high Lp(a) with different types of CVD. In this cross-sectional analysis, about 1 in 5 individuals had Lp(a) levels above the clinical cutoff value of 50 mg/dL. Reporting Lp(a) values for age- and sex-specific categories, we could observe higher Lp(a) levels in men and elderly people. This study also demonstrates robust and independent associations of high Lp(a) levels and various types of CVD. In contrast, results confirmed no association between elevated Lp(a) and a history of VT. These findings were robustly confirmed in sensitivity analyses after propensity score matching.

The epidemiological distribution of Lp(a) levels has varied throughout various European studies. Median Lp(a) levels in the GNHIES98 cohort (15.3 mg/dL) were moderately higher than in other European cohorts. In a regional population-based analysis from Augsburg, Germany, a median Lp(a) of 11.4 mg/dL was determined, with 14% of the participating individuals having Lp(a) concentrations ≥50 mg/dL.^28^ In the Copenhagen General Population study, median levels of Lp(a) were 9.7 mg/dL.^29^ The BiomarCaRE consortium analyzed data of 52,131 participants from 7 prospective population-based cohorts across Europe, showing an overall median Lp(a) level of 8.7 mg/dL (Q1-Q3: 3.9-19.1 mg/dL), with lower Lp(a) levels in Northern compared to Southern European cohorts.^30^ Finally, the Emerging Risk Factors Collaboration analyzed Lp(a) levels in 126,634 participants across 36 prospective studies and found an overall median Lp(a) of 12.6 mg/dL (Q1-Q3: 4.9-32.1 mg/dL).^5^ However, Lp(a) levels reported in other cohorts were similar to ours. The NHANES III, a nationally representative U.S. cohort, reported a median of 14 mg/dL, and Varvel et al reported a median Lp(a) of 17 mg/dL (Q1-Q3: 7-47 mg/dL) with 24.0% of subjects with levels >50 mg/dL.^17^^,^^31^ The differences between these U.S. cohorts could be attributed to the varying study designs (general population vs referral laboratory), inclusion criteria, and hence epidemiological makeup of the analyzed populations. When comparing epidemiological studies, it is important not only to consider the sample selection but also the differences in ethnicity of the selected populations, as several previous studies have shown a significant variability of Lp(a) levels across ethnicities.^9^^,^^17^^,^^32^^,^^33^

In the GNHIES98 population, we found that Lp(a) levels were generally higher in older individuals, with a notable increase in women around 50 years of age. It is generally hypothesized that the mechanisms behind the increase in Lp(a) levels with age, especially in women, are associated with a reduced Lp(a) clearance due to a declining kidney function. Further, lower plasma estradiol, which inhibits apo(a) gene expression, increases Lp(a) levels in women after menopause.^34^ In support of this hypothesis, previous studies have shown that women who received hormone replacement therapy (HRT) had lower Lp(a) concentrations compared to women not receiving HRT.^35^, ^36^, ^37^ Our findings are congruent with results by Simony et al, which also showed a selective increase in Lp(a) serum levels in women around the age of 50 years.^34^

In contrast to most previous cohorts, such as the UK Biobank or Copenhagen General Population Study, our study revealed higher median Lp(a) levels in men than in women across all age groups, whereas most studies have reported higher overall Lp(a) levels in women.^9^^,^^31^^,^^34^ However, the median Lp(a) level in women from GNHIES98 (10.3 mg/dL) is similar to that reported in the Women’s Health Study, which observed a median of 10.6 mg/dL in women aged 38 to 89 years.^37^ Since the Women’s Health Study and our study were conducted prior to the 2000s, it can be speculated that Lp(a) levels may have been influenced by the more frequent use of HRT, which has been associated with a moderate reduction of Lp(a). A follow-up study from the GNHIES98, for example, revealed a considerable decline in HRT use in women between 1998 and 2003.^38^ While our findings may reflect biological or demographic factors specific to our cohort, they warrant further validation in more recent, methodologically standardized datasets.

Our data exhibited higher median Lp(a) levels with increasing age and higher levels in participants with a history of ASCVD compared to healthy participants. Interestingly, among participants with a history of ASCVD, people aged >55 years had a lower median Lp(a) level in comparison to participants ≤55 years (Supplemental Figure 3B). These observations are in line with reports from the Lp(a)HERITAGE study, which also reported higher median Lp(a) levels in younger (<65 years) compared to older (>65 years) ASCVD patients.^39^ Lp(a) likely plays a prominent role in the development of ASCVD at a younger age, while patients with ASCVD and high Lp(a) may be underrepresented in older age groups due to potentially reduced life expectancy.

The estimated CVD risk associated with elevated Lp(a) levels has varied. In an analysis of UK Biobank data, Patel et al reported a linear relationship between Lp(a) and ASCVD with a 11% increase per 50 nmol/L increment.^7^ In our analysis, there was a robust association between Lp(a) and CVD and ASCVD, with the strongest slope increase at Lp(a) levels between 100 and 150 mg/dL. Furthermore, our analysis revealed an association between increasing Lp(a) levels and a history of heart disease, with the strongest link observed at Lp(a) levels between 100 mg/dL and 180 mg/dL. The association between Lp(a) and cerebrovascular disease was less pronounced, likely due to the combination of ischemic and hemorrhagic strokes.

Due to its homology with plasminogen and the presumed potential of apo(a) to inhibit fibrinolysis, the role of Lp(a) in promoting thrombosis has been previously discussed controversially.^40^ Compared to women without a history of VT, we found that women with a history of VT had significantly higher median Lp(a) levels. However, the observed association of Lp(a) and a history of VT did not persist in multivariable analysis. This aligns with other studies, which observed no association between VT and Lp(a), and we therefore regard our findings as confirmatory of the established consensus.^41^ The significantly higher levels in women with a history of VT may be attributed to the overall different makeup of our study population and could potentially be influenced by additional factors such as hormonal status or changes related to menopause. It is generally assumed that Lp(a) predominantly mediates atherosclerotic stenosis, while its role in VT is minor.^41^^,^^42^ Nevertheless, elevated Lp(a) levels are believed to increase the risk of VT when combined with other thrombophilic risk factors, such as factor V Leiden.^43^

### Study strengths and limitations

This study has multiple strengths. First, the analyzed cohort is, to the best of our knowledge, the first to report the Lp(a) distribution in a nationwide, representative German population. Second, the analyses were adjusted for complex sample design, accounting for the cluster sampling and response behavior, thereby providing more accurate and representative conclusions for the German population. Third, the sample size of the study population was sufficient to adjust for various potential confounding factors in our regression analysis including lipid-lowering medication, which is lacking in several previous reports.^9^^,^^17^ Fourth, we were able to investigate distinct associations between Lp(a) levels and different types of CVD and carried out analyses stratified by age and sex. Fifth, our study reports data of a mostly “statin naïve” population, which likely represents true Lp(a) levels of most participants, as the rate of Lp(a)-modifying therapy was very low (<5%).

However, several limitations exist. The data of the GNHIES98 study population were collected between 1997 and 1999; thus, results do not necessarily reflect the current population of Germany, and treatment standards have evolved in the past years. Nevertheless, the reported data serves as an important foundation and proposes Lp(a) testing in other representative studies. The assay used in this study population measured Lp(a) in mass units (mg/L) and does not adhere to current consensus recommendations to quantify Lp(a) in molar concentrations (nmol/L).^4^^,^^44^ Future studies of the German population should be conducted using new Lp(a) testing methodology that is in line with the current standard and measures Lp(a) in molar units to assess potential changes in Lp(a) distribution among the German population. The endpoints of this study were participant-reported outcomes and therefore could have been falsely reported, as the accuracy of self-reported cardiovascular events is variable, potentially reducing the internal validity of the study. However, questionnaire-based, self-reported outcomes are standard practice in large-scale, population-based observational studies. In GNHIES98, the information was further verified through interviews conducted by physicians. In addition, the outcomes did not differentiate between ischemic and hemorrhagic stroke, whereas the association of Lp(a) has primarily been observed with ischemic stroke only.^12^ Although the overall sample size was large, the number of cases reported for each endpoint was rather low in this population, limiting the power of our results. To address this, we created composite outcomes to strengthen the robustness of our findings. Lastly, observational studies by nature are susceptible to biases and confounding, which do not allow us to imply a causal relationship between Lp(a) and CVD.

Our findings provide important insight into the distribution of Lp(a) levels in a representative sample of the German adult population, highlighting that a substantial proportion of individuals exhibit elevated Lp(a) concentrations above the commonly used risk threshold of >50 mg/dL. Compared to data from other European populations, Lp(a) levels appear notably higher in this population, suggesting a potentially greater burden of Lp(a)-related ASCVD risk in Germany. Interestingly, while most large population-based studies have reported higher Lp(a) levels in women, we observed higher levels in men in our analysis. This unexpected sex difference highlights the need to replicate our findings using more recent and standardized data to evaluate whether this pattern persists in the contemporary German population.

While our data are based on measurements from the late 1990s, they offer a valuable foundation for informing population-specific screening strategies aimed at earlier identification of individuals at increased ASCVD risk due to elevated Lp(a) in Germany. Given that Lp(a) is largely genetically determined and hardly modifiable by lifestyle factors, identifying high-risk individuals early may be particularly relevant for personalized prevention strategies, including emerging Lp(a)-lowering therapies. However, we acknowledge that our findings should be replicated in more recent and methodologically updated datasets to confirm the persistently high Lp(a) burden in the German population. Such contemporary data would be essential to refine risk estimation and guide the implementation of evidence-based screening strategies tailored specifically to the German context.

## Conclusions

In this nationally representative German cohort, 1 in 5 individuals had Lp(a) levels exceeding 50 mg/dL. Among this largely statin-naïve population, Lp(a) concentrations were consistently higher in men than women across all age groups and were independently associated with a history of ASCVD, particularly in older individuals and at high concentrations. No association was found between Lp(a) and VT. Updated health surveys using new testing methods are needed to prospectively assess the role of Lp(a) in CVD risk in Germany.

Perspectives **COMPETENCY IN MEDICAL KNOWLEDGE:** Lp(a) is a significant risk factor for ASCVD with elevated levels observed in about 1 in 5 individuals in the general German population.

**TRANSLATIONAL OUTLOOK:** Updated population-based studies are essential to clarify the role Lp(a) in determining lifetime CVD risk. Incorporating Lp(a) into refined risk prediction models could guide targeted prevention strategies and improve population-level outcomes, particularly as new treatment options become available.

## Funding support and author disclosures

The original GNHIES98 survey was supported by the German Federal Ministry of Health. Dr Endres has received funding from DFG under Germany’s Excellence Strategy–EXC-2049 to 390688087, Collaborative Research Center ReTune TRR 295 to 424778381, Clinical Research Group KFO 5023 BeCAUSE-Y, project 2 EN343/16-1, BMBF, DZNE, DZHK, DZPG, EU, Corona Foundation, and Fondation Leducq. Dr Nave has received funding from the 10.13039/100016021Corona Foundation, the German Center for Cardiovascular Research (DZHK), and the 10.13039/501100003042Else Kröner-Fresenius-Stiftung (EKFS); and has received honoraria from Novartis, Pvzer/BMS, and Ipsen Pharma, all outside the submitted work. Dr Endres has received grants from Bayer and fees paid to the Charité from Amgen, AstraZeneca, Bayer Healthcare, Boehringer Ingelheim, BMS, Daiichi Sankyo, Sanofi, and Pfizer, all outside the submitted work. All other authors have reported that they have no relationships relevant to the contents of this paper to disclose.
