# Association of Lipoprotein(a) With Cardiovascular and Cerebrovascular Disease in a Nationally Representative Cohort of Germany

**DOI:** 10.1016/j.jacadv.2025.102533

**Published:** 2026-01-28

Gelfert GG, Grittner U, Kuhnert R, Scheidt-Nave C, Endres M, Nave AH

Association of Lipoprotein(a) With Cardiovascular and Cerebrovascular Disease in a Nationally Representative Cohort of Germany

*JACC Adv*. 2025;4:102015.

During a post-publication review of the results, the authors identified that 2 tables in the main paper and 4 tables in the Supplemental Appendix do not reflect the full multiple regression model. Specifically, the variable *lipid-lowering medication* was inadvertently omitted from these tables. As a result, the parameter estimates shown in the affected tables do not correspond to the fully specified model.

Importantly, the text and all figures in the published paper are based on the correct model including *lipid-lowering medication*, and therefore the scientific interpretation in the paper is accurate.

The authors have re-run all analyses using the complete model and confirmed that the overall conclusions and interpretations of the paper remain unchanged. While several numerical estimates in the tables change slightly when including lipid-lowering medication, the text of the paper does not need to be modified.

The current online version of the paper includes the updated tables and Supplemental Appendix.

The authors apologize for these errors.

