# Supplementary material for: Association of Lipoprotein(a) With Cardiovascular and Cerebrovascular Disease in a Nationally Representative Cohort of Germany
Source: JACC Adv. 2025 Jul 23;4(8):102015. doi: 10.1016/j.jacadv.2025.102015 (PMC12309279; doi:10.1016/j.jacadv.2025.102015)
Supplement: Supplementary Material [file mmc1.pdf]

**Supplemental Table 1.** Median Lp(a) stratified by demographics, history of cardiovascular disease, and cardiovascular risk factors, GNHIES98.

| Covariables (total n/n=missing*) | Lp(a) in mg/dL, median (IQR) | P-Value | SMD |
|---|---|---|---|
| **Age groups (6,657/0)** | | <0.001 | 0.243 |
| ≤ 55 (n=4571) | 13.3 (5.6-39.0) | | |
| > 55 (n=2086) | 20.5 (6.9-52.1) | | |
| **Sex (6,657/0)** | | <0.001 | 0.209 |
| Female (n=3443) | 10.3 (5.6-34.0) | | |
| Male (n=3214) | 22.1 (7.4-51.3) | | |
| **Arterial hypertension (6,622/35)** | | <0.001 | 0.187 |
| Yes (n=1890) | 20.9 (6.9-51.6) | | |
| No (n=5368) | 13.6 (5.6-40.0) | | |
| **Diabetes mellitus (6,633/24)** | | <0.006 | 0.127 |
| Yes (n=336) | 22.2 (7.4-48.5) | | |
| No (n=6297) | 15.0 (5.6-42.4) | | |
| **Dyslipidemia (6,633/24)** | | <0.001 | 0.874 |
| Yes (n=1463) | 26.0 (8.2-61.0) | | |
| No (n=5170) | 13.3 (5.6-38.1) | | |
| **Smoking (6,501/156)** | | 0.002 | 0.069 |
| Never (n=2958) | 13.3 (5.6-40.2) | | |
| Former (n=1412) | 18.0 (5.6-45.5) | | |

| | | | |
|---|---|---|---|
| Active (n=2131) | 16.3 (5.6-44.8) | | |
| **Alcohol consumption (6,468/189)** | | <0.001 | 0.073 |
| Never (n=1157) | 15.4 (5.6-44.8) | | |
| Moderate (n=4069) | 14.2 (5.6-40.7) | | |
| Heavy (n=1242) | 18.6 (5.8-47.7) | | |
| **Coronary Heart Disease (6,633/24)** | | <0.001 | 0.273 |
| Yes (n=382) | 23.1 (8.2-61.7) | | |
| No (n=6251) | 14.9 (5.6-42.1) | | |
| **Heart Failure (6,633/24)** | | <0.001 | 0.383 |
| Yes (n=209) | 28.3 (7.6-66.0) | | |
| No (n=6424) | 15.0 (5.6-42.1) | | |
| **Cerebral Circulatory Disorder (6,633/24)** | | 0.021 | 0.340 |
| Yes (n=113) | 22.9 (5.8-65.9) | | |
| No (n=6520) | 15.1 (5.6-42.7) | | |
| **Circulatory Disorder of the Legs (6,633/24)** | | 0.001 | 0.307 |
| Yes (n=176) | 22.8 (7.6-58.4) | | |
| No (n=6457) | 15.0 (5.6-42.5) | | |
| **Venous Thrombosis (6,633/24)** | | 0.106 | 0.138 |
| Yes (n=263) | 19.2 (6.9-46.3) | | |
| No (n=6370) | 15.1 (5.6-42.9) | | |
| **Myocardial Infarction (6,633/24)** | | <0.001 | 0.301 |
| Yes (n=147) | 27.4 (11.1-57.9) | | |

| | | | |
|---|---|---|---|
| No (n=6486) | 15.0 (5.6-42.8) | | |
| **Stroke (6,633/24)** | | 0.021 | 0.333 |
| Yes (n=89) | 23.3 (7.1-65.6) | | |
| No (n=6544) | 15.2 (5.6-42.6) | | |
| **Heart disease (6,633/24)** | | <0.001 | 0.311 |
| Yes (n=508) | 24.4 (7.9-64.0) | | |
| No (n=6125) | 14.7 (5.6-41.3) | | |
| **Cerebrovascular disease (6,633/24)** | | 0.010 | 0.276 |
| Yes (n=164) | 22.5 (5.8-63.3) | | |
| No (n=6469) | 15.1 (5.6-42.4) | | |
| **ASCVD (6,633/24)** | | <0.001 | 0.299 |
| Yes (n=712) | 23.6 (7.1-63.0) | | |
| No (n=5921) | 14.5 (5.6-40.7) | | |
| **CVD (6,633/24)** | | <0.001 | 0.262 |
| Yes (n=869) | 22.5 (7.1-58.6) | | |
| No (n=5764) | 14.3 (5.6-40.7) | | |

* Total number of observations varies according to the number of missing observations for covariables.

Results from bivariable analyses, weighted for the German population structure as of December 31st, 1997, not adjusted for complex study design. n = unweighted and unadjusted sample size.

SMD=Standardized median difference.

**CVD:** History of any cardiovascular disease including self-reported history of myocardial infarction, coronary heart disease, stroke, cerebral circulatory disorder, peripheral artery disease, heart failure or venous thrombosis

**ASCVD:** History of any atherosclerotic cardiovascular disease including self-reported history of myocardial infarction, coronary heart disease, stroke, cerebral circulatory disorder or peripheral artery disease

**Supplemental Table 2.** Proportion of Lp(a) levels ≥50 mg/dL stratified by demographics, history of cardiovascular disease, and cardiovascular risk factors, GNHIES98.

| | | **Lp(a) ≥50 mg/dL, % (n)** | **P-Value** |
|---|---|---|---|
| **N** | | **21.6% (1385)** | |
| Age Groups | Age <55years | 19.7% (872) | <0.001 |
| | Age ≥55years | 25.9% (513) | |
| Sex | Male | 25.7% (791) | <0.001 |
| | Female | 17.6% (594) | |
| **Cardiovascular risk factors** | | | |
| Arterial hypertension | Yes | 25.6% (472) | <0.001 |
| | No | 20.0% (904) | |
| Diabetes mellitus | Yes | 24.7% (83) | 0.236 |
| | No | 21.4% (1294) | |
| Dyslipidemia | Yes | 31.0% (435) | <0.001 |
| | No | 18.8% (942) | |
| BMI ≥25kg/m$^2$ | Yes | 26.2% (1010) | <0.001 |
| | No | 14.4% (369) | |
| BMI ≥30kg/m$^2$ | Yes | 27.1% (372) | <0.001 |
| | No | 20.1% (1007) | |
| Ever Smoker | Yes | 22.6% (782) | 0.047 |
| | No | 20.3% (569) | |
| Heavy alcohol consumption | Yes | 23.6% (295) | 0.200 |
| | No | 21.4% (1047) | |
| **History of cardiovascular diseases** | | | |

| | | | |
|---|---|---|---|
| CVD | Yes | 28.8% (235) | <0.001 |
| | No | 20.4% (1142) | |
| ASCVD | Yes | 31.0% (208) | <0.001 |
| | No | 20.4% (1169) | |
| Heart disease | Yes | 30.8% (140) | <0.001 |
| | No | 20.8% (1237) | |
| Cerebrovascular disease | Yes | 33.2% (54) | <0.001 |
| | No | 21.3% (1323) | |
| Coronary heart disease | Yes | 30.2% (107) | <0.001 |
| | No | 21.0% (1270) | |
| Myocardial infarction | Yes | 27.3% (43) | 0.175 |
| | No | 21.4% (1334) | |
| Heart failure | Yes | 33.6% (59) | 0.002 |
| | No | 21.1% (1318) | |
| Cerebral circulatory disorder | Yes | 35.1% (39) | 0.001 |
| | No | 21.3% (1338) | |
| Stroke | Yes | 36.6% (30) | 0.003 |
| | No | 21.3% (1347) | |
| PAD | Yes | 29.4% (49) | 0.038 |
| | No | 21.3% (1328) | |
| Venous thrombosis | Yes | 22.0% (58) | 0.865 |
| | No | 21.5% (1319) | |
| **Medication** | | | |
| Lipid lowering therapy | Yes | 39.2% (110) | <0.001 |

| | | | |
|---|---|---|---|
| | No | 20.7% (1266) | |
| Antihypertensive medication | Yes | 26.1% (288) | <0.001 |
| | No | 20.7% (1088) | |
| Glucose lowering therapy | Yes | 26.6% (59) | 0.137 |
| | No | 21.3% (1316) | |

Results from bivariable analyses, weighted for the German population structure as of December 31st, 1997, adjusted for complex study design. n = unweighted and unadjusted sample size.

**CVD:** History of any cardiovascular disease including self-reported history of myocardial infarction, coronary heart disease, stroke, cerebral circulatory disorder, peripheral artery disease, heart failure or venous thrombosis.

**ASCVD:** History of any atherosclerotic cardiovascular disease including self-reported history of myocardial infarction, coronary heart disease, stroke, cerebral circulatory disorder or peripheral artery disease.

**Heart disease**: History of acute myocardial infarction, chronic coronary heart disease or heart failure.

**Cerebrovascular disease:** History of stroke or cerebral circulatory disorder.

**Supplemental Table 3.** Results from spline analysis of the independent association of Lp(a) cut-points with history of CVD, GNHIES98.

| Lp(a) | Heart disease (n=397) | | Cerebrovascular Disease (n=152) | | PAD (n=164) | | Heart failure (n=200) | |
|---|---|---|---|---|---|---|---|---|
| | OR* | 95% CI | OR* | 95% CI | OR* | 95% CI | OR* | 95% CI |
| 15mg/dL | 1 | | 1 | | 1 | | 1 | |
| 30mg/dL | 1.00 | 0.90-1.12 | 1.01 | 0.85-1.21 | 0.97 | 0.82-1.13 | 0.97 | 0.83-1.13 |
| 50mg/dL | 1.06 | 0.89-1.26 | 1.07 | 0.80-1.42 | 0.95 | 0.73-1.23 | 1.06 | 0.83-1.36 |
| 70mg/dL | 1.17 | 0.95-1.44 | 1.16 | 0.83-1.61 | 0.96 | 0.70-1.31 | 1.28 | 0.97-1.70 |
| 100mg/dL | 1.42 | 1.07-1.88 | 1.35 | 0.86-2.11 | 1.07 | 0.71-1.60 | 1.82 | 1.25-2.65 |
| 150mg/dL | 1.77 | 1.21-2.59 | 1.71 | 0.95-3.09 | 1.71 | 1.00-2.93 | 2.42 | 1.48-3.94 |
| 180mg/dL | 1.69 | 1.03-2.77 | 1.83 | 0.88-3.83 | 2.86 | 1.60-5.12 | 1.77 | 0.89-3.52 |

| Lp(a) | MI (n=137) | | Coronary Heart Disease (n=359) | | Stroke (n=78) | | Cerebral Circulatory Disorder (n=106) | |
|---|---|---|---|---|---|---|---|---|
| | OR* | 95% CI | OR* | 95% CI | OR* | 95% CI | OR* | 95% CI |
| 15mg/dL | 1 | | 1 | | 1 | | 1 | |
| 30mg/dL | 1.06 | 0.87-1.29 | 0.94 | 0.83-1.07 | 1.03 | 0.80-1.31 | 0.98 | 0.80-1.21 |
| 50mg/dL | 1.07 | 0.78-1.47 | 0.96 | 0.78-1.17 | 1.12 | 0.76-1.66 | 1.03 | 0.73-1.45 |
| 70mg/dL | 1.02 | 0.70-1.49 | 1.06 | 0.84-1.34 | 1.28 | 0.81-2.01 | 1.14 | 0.77-1.70 |
| 100mg/dL | 0.94 | 0.57-1.54 | 1.30 | 0.94-1.82 | 1.61 | 0.89-2.91 | 1.44 | 0.86-2.39 |
| 150mg/dL | 0.99 | 0.51-1.92 | 1.48 | 0.96-2.30 | 2.18 | 1.01-4.68 | 2.18 | 1.12-4.23 |
| 180mg/dL | 1.36 | 0.64-2.87 | 1.10 | 0.60-2.03 | 2.23 | 0.84-5.90 | 2.57 | 1.20-5.53 |

Results weighted for the German population structure as of December 31st, 1997; not adjusted for complex study design. n = unweighted and unadjusted sample size.

* OR: Marginal odds ratios from multivariable regression of serum Lp(a) with splines on history of CVD adjusted for sex, age, dyslipidemia, arterial hypertension, diabetes mellitus, BMI risk categories, smoking (ever), lipid lowering medication.

Analyses are based on 6,435 individuals with complete data.

**CVD:** History of any cardiovascular disease including self-reported myocardial infarction, coronary heart disease, stroke, cerebral circulatory disorder, peripheral artery disease, heart failure or venous thrombosis.

**ASCVD:** History of any atherosclerotic cardiovascular disease including self-reported myocardial infarction, coronary heart disease, heart failure, stroke, cerebral circulatory disorder or peripheral artery disease.

**Heart disease**: History of acute myocardial infarction, chronic coronary heart disease or heart failure.

**Cerebrovascular disease:** History of stroke or cerebral circulatory disorder.

**Supplemental Table 4.** Independent association of multiple dichotomous Lp(a) cut-points with history of CVD in the GNHIES98.

| Covariate | CVD (n=857) OR* (95% CI) | CVD P-value | ASCVD (n=674) OR* (95% CI) | ASCVD P-value | Venous Thrombosis (n=252) OR* (95% CI) | Venous Thrombosis P-value |
|---|---|---|---|---|---|---|
| Lp(a) ≥30mg/dl | 1.20 (0.96, 1.49) | 0.302 | 1.20 (0.96, 1.49) | 0.109 | 0.82 (0.59, 1.13) | 0.228 |
| Lp(a) ≥100mg/dl | 1.42 (1.03, 1.95) | 0.033 | 1.48 (1.08, 2.05) | 0.017 | 1.30 (0.71, 2.38) | 0.396 |

| Covariate | Heart Disease (n=397) OR* (95% CI) | Heart Disease P-value | Cerebrovascular Disease (n=152) OR* (95% CI) | Cerebrovascular Disease P-value | PAD (n=164) OR* (95% CI) | PAD P-value |
|---|---|---|---|---|---|---|
| Lp(a) ≥30mg/dl | 1.20 (0.91, 1.59) | 0.194 | 1.21 (0.82, 1.77) | 0.336 | 1.07 (0.70, 1.63) | 0.747 |
| Lp(a) ≥50mg/dl | 1.28 (0.94, 1.74) | 0.122 | 1.50 (1.00, 2.24) | 0.049 | 1.23 (0.76, 2.00) | 0.392 |
| Lp(a) ≥100mg/dl | 1.46 (0.98, 2.18) | 0.062 | 1.54 (0.92, 2.59) | 0.101 | 1.84 (1.10, 3.05) | 0.020 |

Results weighted for the German population structure as of December 31st, 1997; adjusted for complex study design. n = unweighted and unadjusted sample size.

* OR = odds ratios from multivariable logistic regression models adjusted for sex, age, dyslipidemia, arterial hypertension, diabetes mellitus, BMI risk categories, smoking (ever), lipid lowering medication.

Analyses are based on = 6,435 individuals with complete data.

**CVD**: History of any cardiovascular disease including self-reported myocardial infarction, coronary heart disease, stroke, cerebral circulatory disorder, peripheral artery disease, heart failure or venous thrombosis.

**ASCVD:** History of any atherosclerotic cardiovascular disease including self-reported myocardial infarction, coronary heart disease, heart failure, stroke, cerebral circulatory disorder or peripheral artery disease.

**Heart disease**: History of acute myocardial infarction, chronic coronary heart disease or heart failure.

**Cerebrovascular disease:** History of stroke or cerebral circulatory disorder.

**Supplement Table 5.** Marginal means* for Lipoprotein(a) (bias-adjusted, back-transformed)

| | Response (95% CI) |
|---|---|
| **History of CVD** | |
| No (n=2,209) | 30.5 (28.9, 32.1) |
| Yes (n=786) | 34.0 (31.4, 36.8) |
| **History of ASCVD** | |
| No (n=1,818) | 31.6 (29.9, 33.4) |
| Yes (n=642) | 35.7 (32.7, 38.9) |
| **History of Venous thrombosis** | |
| No (n=1,215) | 32.8 (30.5, 35.3) |
| Yes (n=251) | 32.9 (28.6, 37.9) |

*Marginal means were estimated with linear mixed-effects models using log-transformed Lp(a) as the dependent variable, with age, sex, their interaction, and event status (yes/no) as independent variables. Random intercepts for matching groups were included.

**CVD**: History of any cardiovascular disease including self-reported myocardial infarction, coronary heart disease, stroke, cerebral circulatory disorder, peripheral artery disease, heart failure or venous thrombosis.

**ASCVD:** History of any atherosclerotic cardiovascular disease including self-reported myocardial infarction, coronary heart disease, heart failure, stroke, cerebral circulatory disorder or peripheral artery disease.

**Supplement Table 6.** Pairwise comparisons of marginal means* for Lipoprotein(a) (back-transformed to response scale)

| Contrast | Ratio (95% CI) | P-value |
|---|---|---|
| History of CVD No (n=2,209)/ Yes (n=786) | 0.90 (0.82, 0.98) | 0.015 |
| History of ASCVD No (n=1,818) / Yes (n=642) | 0.88 (0.80, 0.98) | 0.014 |
| History of Venous thrombosis No (n=1,215) / Yes (n=251) | 1.00 (0.86, 1.16) | 0.972 |

*Marginal means were estimated with linear mixed-effects models using log-transformed Lp(a) as the dependent variable, with age, sex, their interaction, and event status (yes/no) as independent variables. Random intercepts for matching groups were included.

**CVD**: History of any cardiovascular disease including self-reported myocardial infarction, coronary heart disease, stroke, cerebral circulatory disorder, peripheral artery disease, heart failure or venous thrombosis.

**ASCVD:** History of any atherosclerotic cardiovascular disease including self-reported myocardial infarction, coronary heart disease, heart failure, stroke, cerebral circulatory disorder or peripheral artery disease.

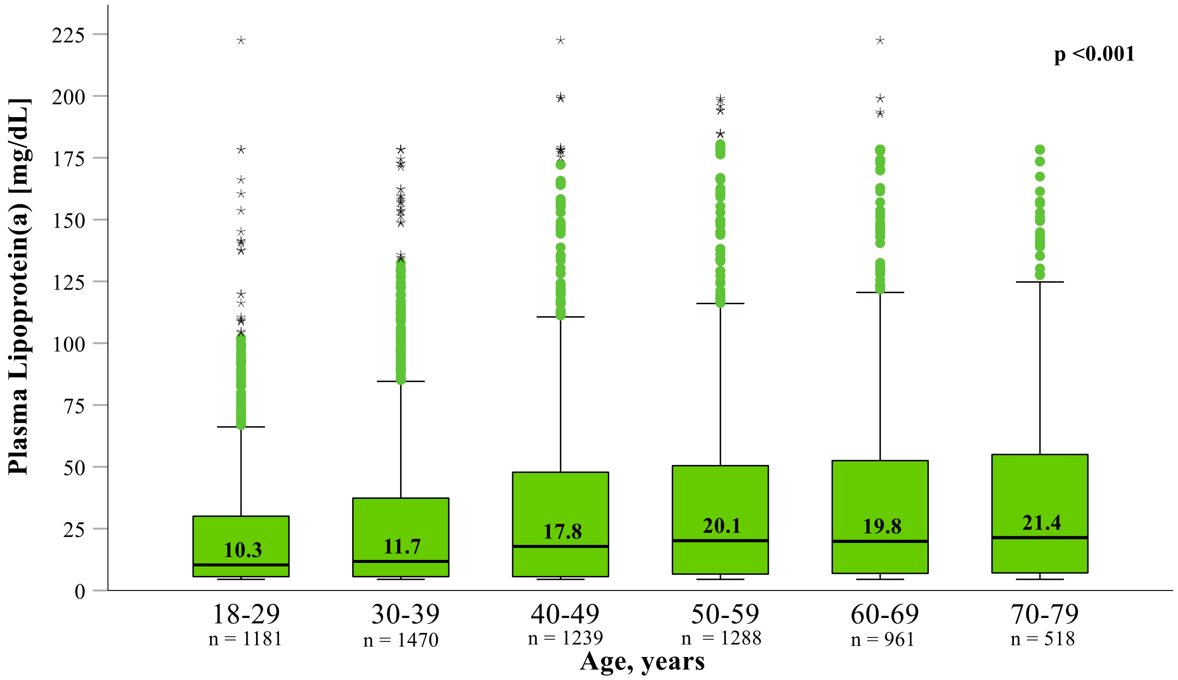


**Supplemental Figure 1.** Median lipoprotein(a) levels across age categories among adults in Germany.

Caption: Analyses were based on 6,657 individuals (aged 18-79 years) from the German National Health Interview and Examination Survey 1998.

Bars indicate median and interquartile range. *p* values are calculated with Kruskal-Wallis-Test.

**Figure 2A**

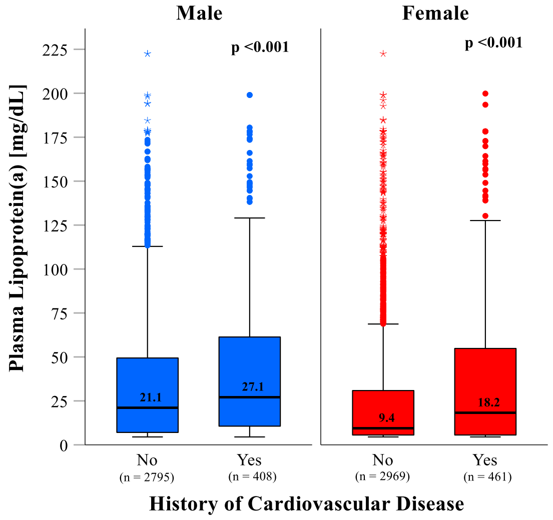


**Figure 2B**

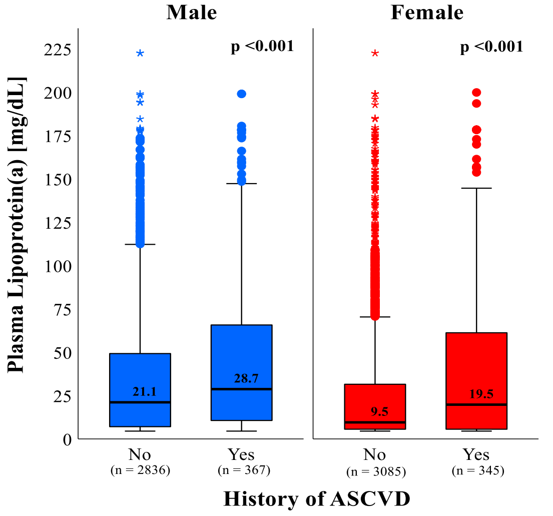


**Figure 2C**

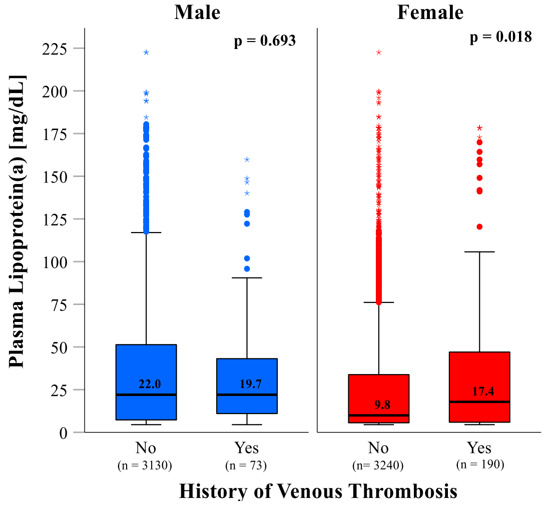


**Figure 2D**

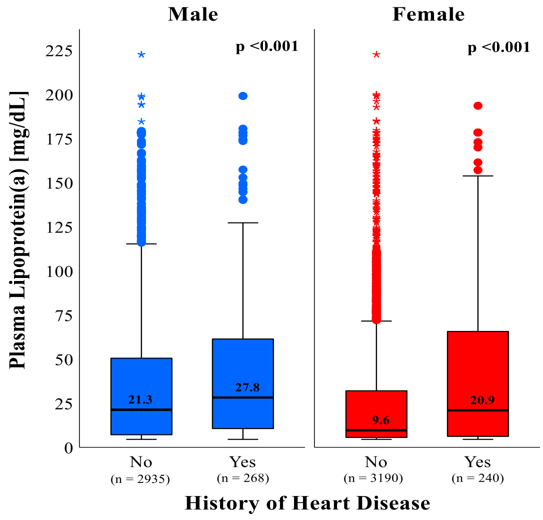


**Figure 2E**

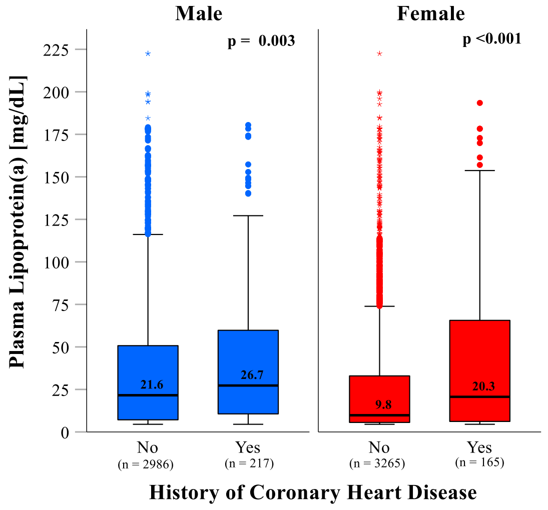


**Figure 2F**

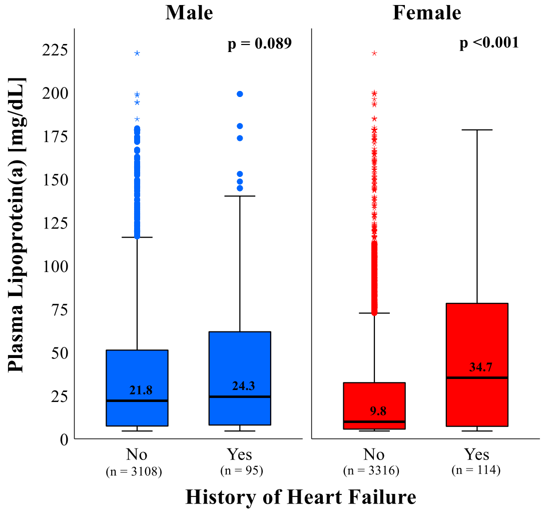

**Figure 2G**

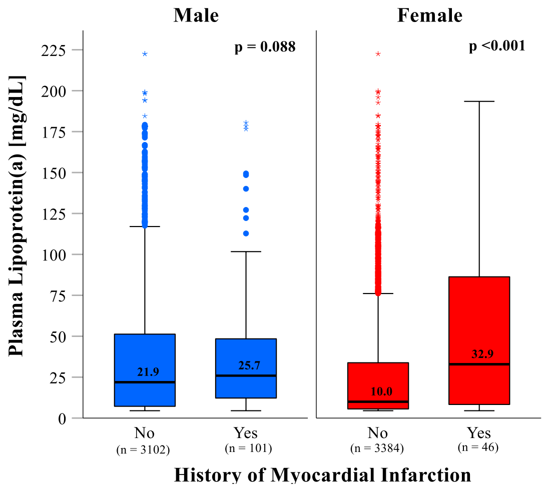


**Figure 2H**

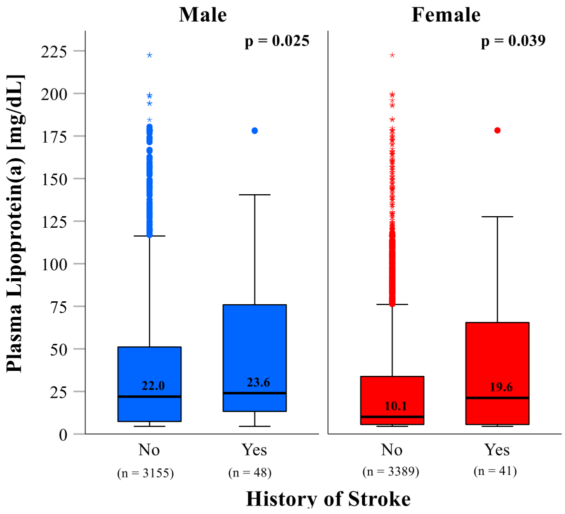


**Figure 2I**

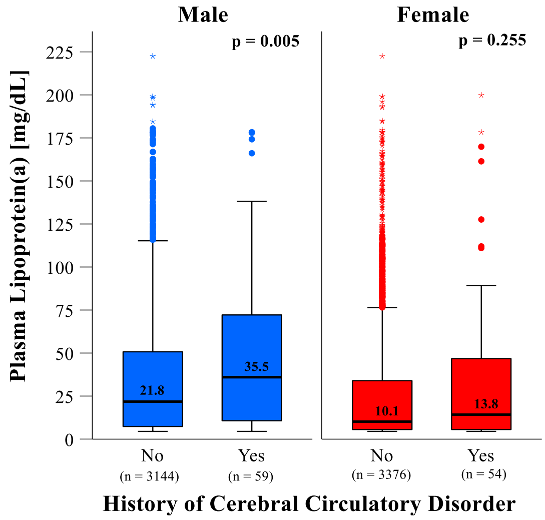


**Figure 2J**

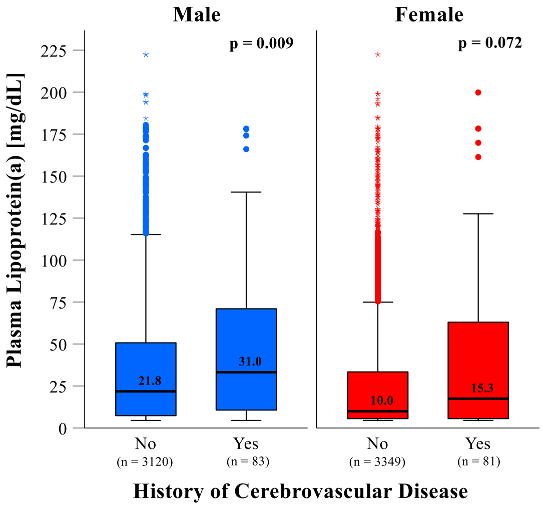


**Figure 2K**

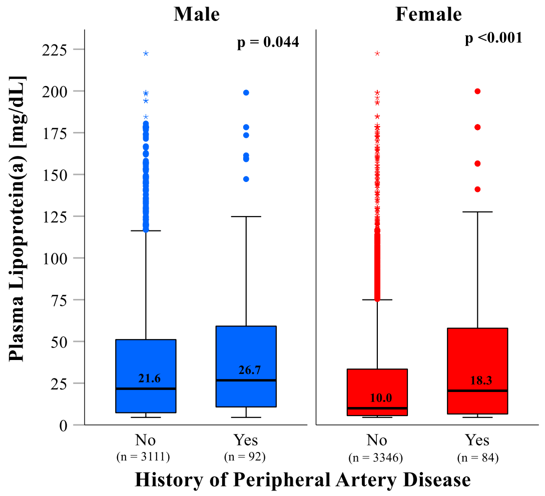

**Supplemental Figure 2.** Median lipoprotein(a) by history of cardiovascular disease and sex.

Caption: Analyses were based on 6,633 individuals (aged 18-79 years) with complete data from the German National Health Interview and Examination Survey 1998. Women: n=3,430, men: n=3,203. Bars indicate median and interquartile range. *p* values are calculated with Mann-Whitney-U-Test.

**Figure 3A**

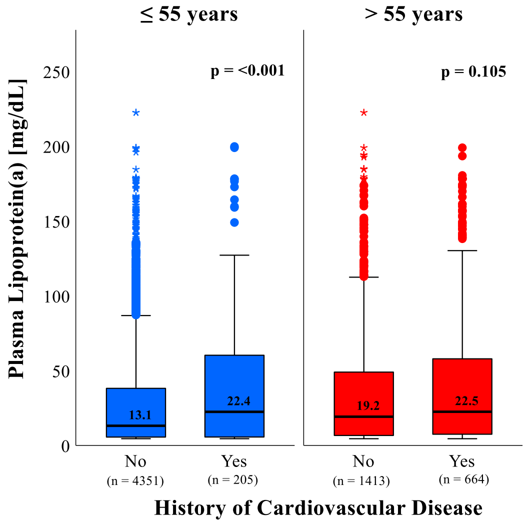


**Figure 3B**

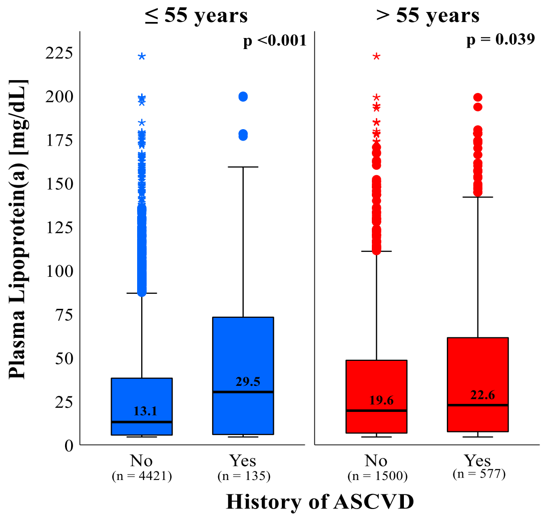


**Figure 3C**

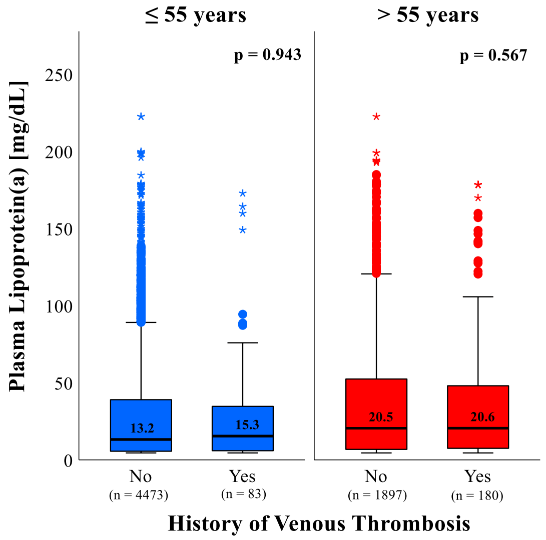


**Supplemental Figure 3.** Median lipoprotein(a) by history of cardiovascular disease stratified by age groups.

Caption: Analyses were based on 6,633 individuals (aged 18-79 years) with complete data from the German National Health Interview and Examination Survey 1998. Age ≤55y n=4556; age >55y =2077. Bars indicate median and interquartile range. *p* values are calculated with Mann-Whitney-U-Test.

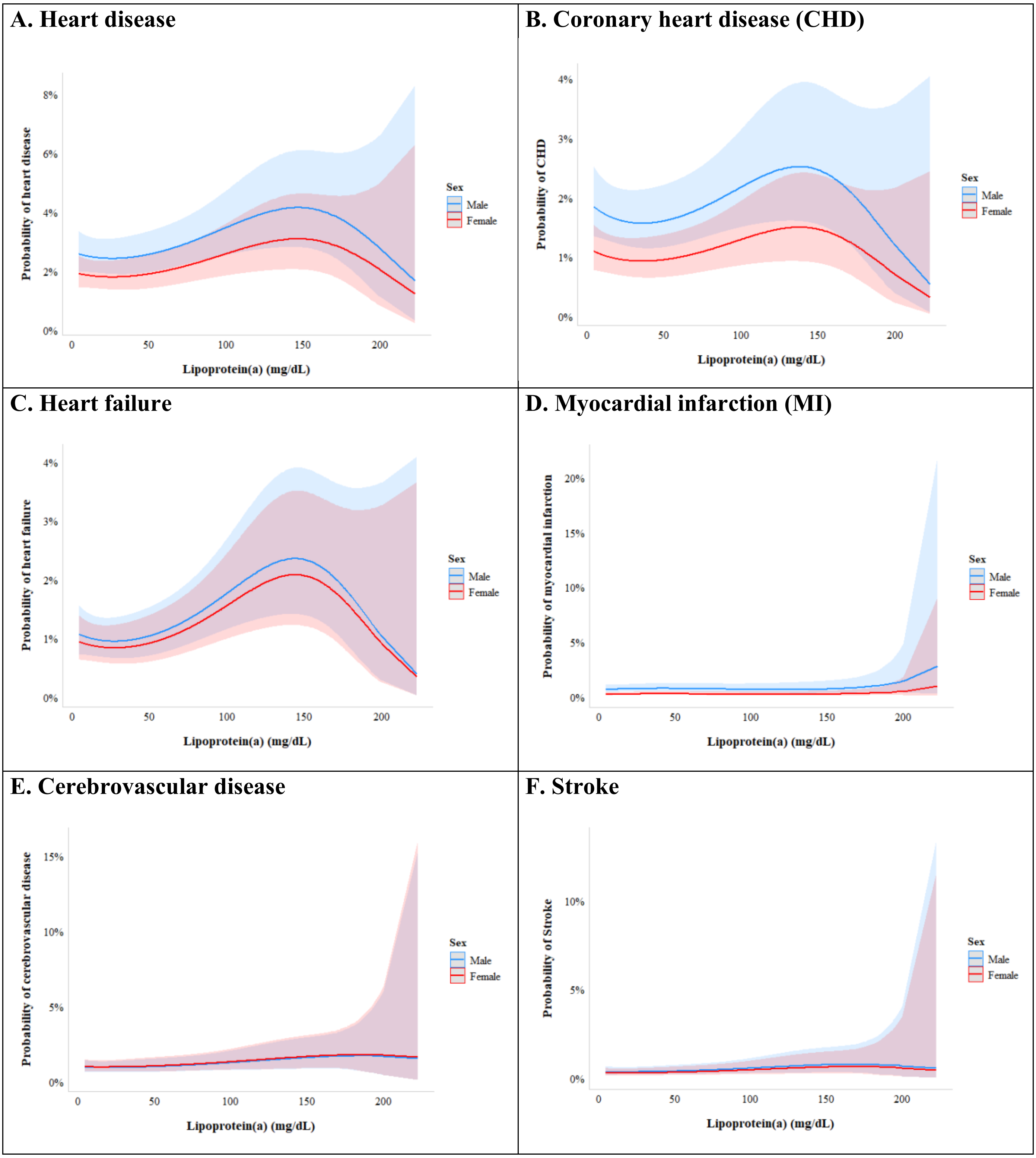
A. Heart disease
Probability of heart disease
0%
2%
4%
6%
8%
0
50
100
150
200
Lipoprotein(a) (mg/dL)
Sex
Male
Female
B. Coronary heart disease (CHD)
Probability of CHD
0%
1%
2%
3%
4%
0
50
100
150
200
Lipoprotein(a) (mg/dL)
Sex
Male
Female
C. Heart failure
Probability of heart failure
0%
1%
2%
3%
4%
0
50
100
150
200
Lipoprotein(a) (mg/dL)
Sex
Male
Female
D. Myocardial infarction (MI)
Probability of myocardial infarction
0%
5%
10%
15%
20%
0
50
100
150
200
Lipoprotein(a) (mg/dL)
Sex
Male
Female
E. Cerebrovascular disease
Probability of cerebrovascular disease
0%
5%
10%
15%
0
50
100
150
200
Lipoprotein(a) (mg/dL)
Sex
Male
Female
F. Stroke
Probability of Stroke
0%
5%
10%
0
50
100
150
200
Lipoprotein(a) (mg/dL)
Sex
Male
Female

**G. Cerebral circulatory disorder**

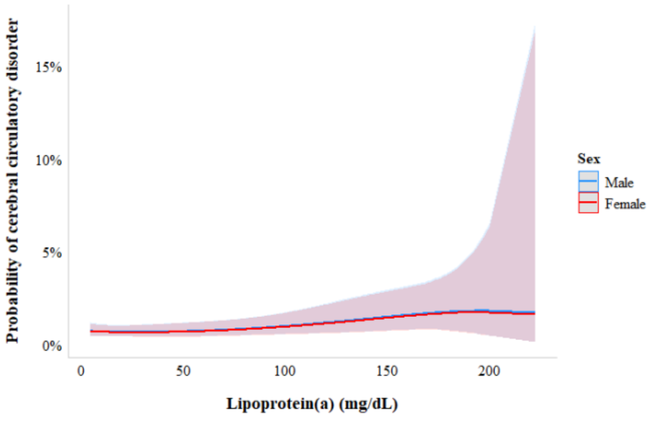


**I. Peripheral artery disease (PAD)**

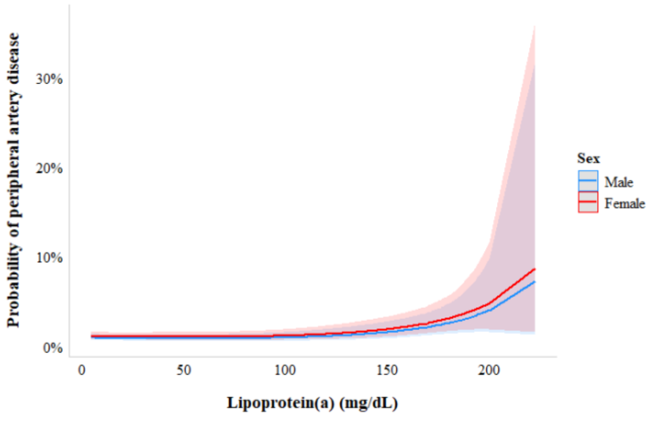


**Supplemental Figure 4.** Spline analysis of the association between CVD and lipoprotein(a) by sex.

Caption: Analyses are based on 6,435 individuals (aged 18-79 years) with complete data from the German National Health Interview and Examination Survey 1998.

Women: n=3,302; men: n=3,133.

**CVD:** History of any cardiovascular disease including self-reported history of myocardial infarction, coronary heart disease, stroke, cerebral circulatory disorder, peripheral artery disease, heart failure or venous thrombosis.

**ASCVD:** History of any atherosclerotic cardiovascular disease including self-reported history of myocardial infarction, coronary heart disease, stroke, cerebral circulatory disorder or peripheral artery disease.

**Heart disease**: History of acute myocardial infarction, chronic coronary heart disease or heart failure.

**Cerebrovascular disease:** History of stroke or cerebral circulatory disorder.

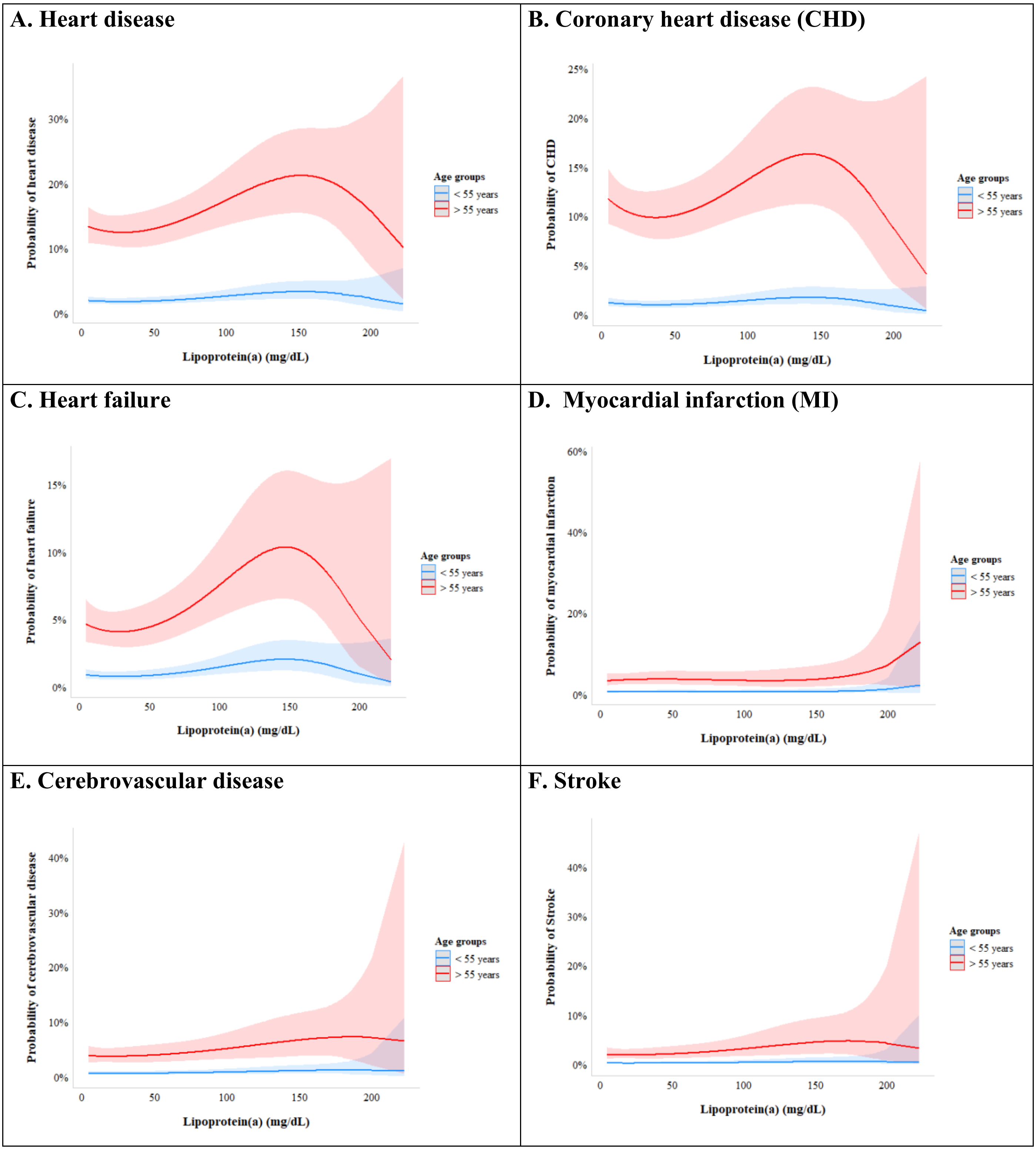
A. Heart disease
Probability of heart disease
0%
10%
20%
30%
Lipoprotein(a) (mg/dL)
0
50
100
150
200
Age groups
< 55 years
> 55 years
B. Coronary heart disease (CHD)
Probability of CHD
0%
5%
10%
15%
20%
25%
Lipoprotein(a) (mg/dL)
0
50
100
150
200
Age groups
< 55 years
> 55 years
C. Heart failure
Probability of heart failure
0%
5%
10%
15%
Lipoprotein(a) (mg/dL)
0
50
100
150
200
Age groups
< 55 years
> 55 years
D. Myocardial infarction (MI)
Probability of myocardial infarction
0%
20%
40%
60%
Lipoprotein(a) (mg/dL)
0
50
100
150
200
Age groups
< 55 years
> 55 years
E. Cerebrovascular disease
Probability of cerebrovascular disease
0%
10%
20%
30%
40%
Lipoprotein(a) (mg/dL)
0
50
100
150
200
Age groups
< 55 years
> 55 years
F. Stroke
Probability of Stroke
0%
10%
20%
30%
40%
Lipoprotein(a) (mg/dL)
0
50
100
150
200
Age groups
< 55 years
> 55 years

**G. Cerebral circulatory disorder**

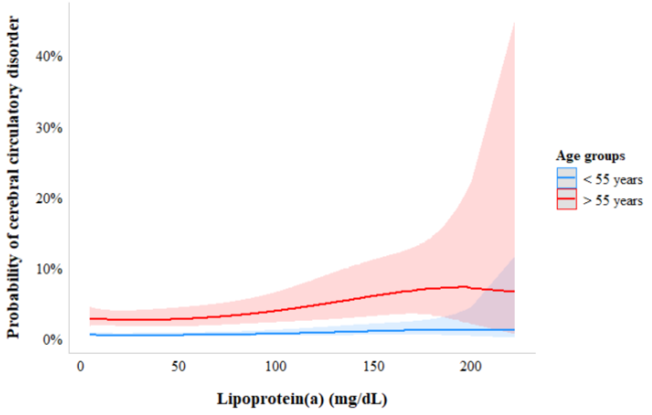


**I. Peripheral artery disease (PAD)**

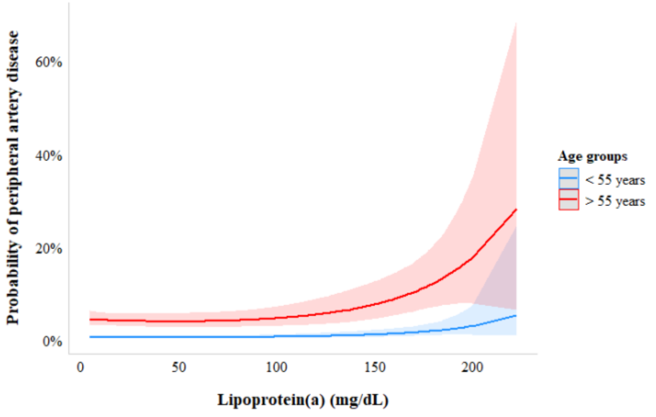


**Supplemental Figure 5.** Spline analysis of the association between CVD and lipoprotein(a) by age groups.

Caption: Analyses are based on 6,435 individuals (aged 18-79 years) with complete data from the German National Health Interview and Examination Survey 1998.

Age ≤55years: 4,444; age >55 years 1,991.

**CVD:** History of any cardiovascular disease including self-reported history of myocardial infarction, coronary heart disease, stroke, cerebral circulatory disorder, peripheral artery disease, heart failure or venous thrombosis.

**ASCVD:** History of any atherosclerotic cardiovascular disease including self-reported history of myocardial infarction, coronary heart disease, stroke, cerebral circulatory disorder or peripheral artery disease.

**Heart disease**: History of acute myocardial infarction, chronic coronary heart disease or heart failure.

**Cerebrovascular disease:** History of stroke or cerebral circulatory disorder.

A. CVD

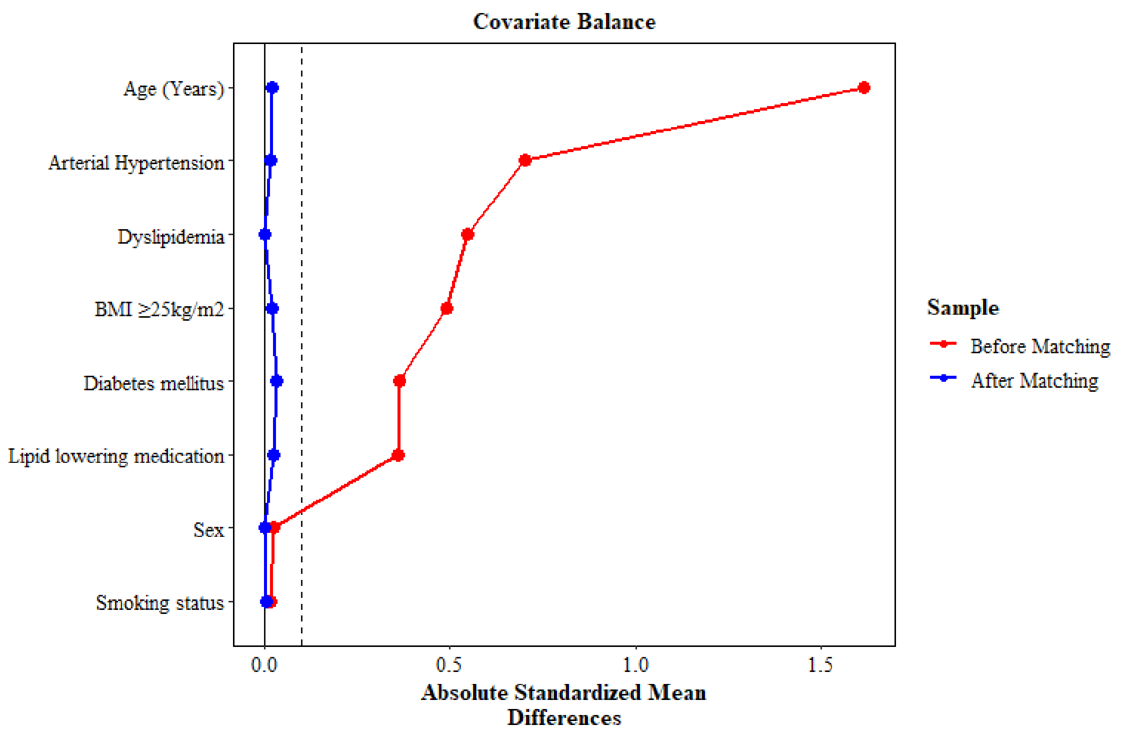
Covariate Balance
Age (Years)
Arterial Hypertension
Dyslipidemia
BMI ≥25kg/m2
Diabetes mellitus
Lipid lowering medication
Sex
Smoking status
0.0
0.5
1.0
1.5
Absolute Standardized Mean Differences
Sample
Before Matching
After Matching

B. ASCVD

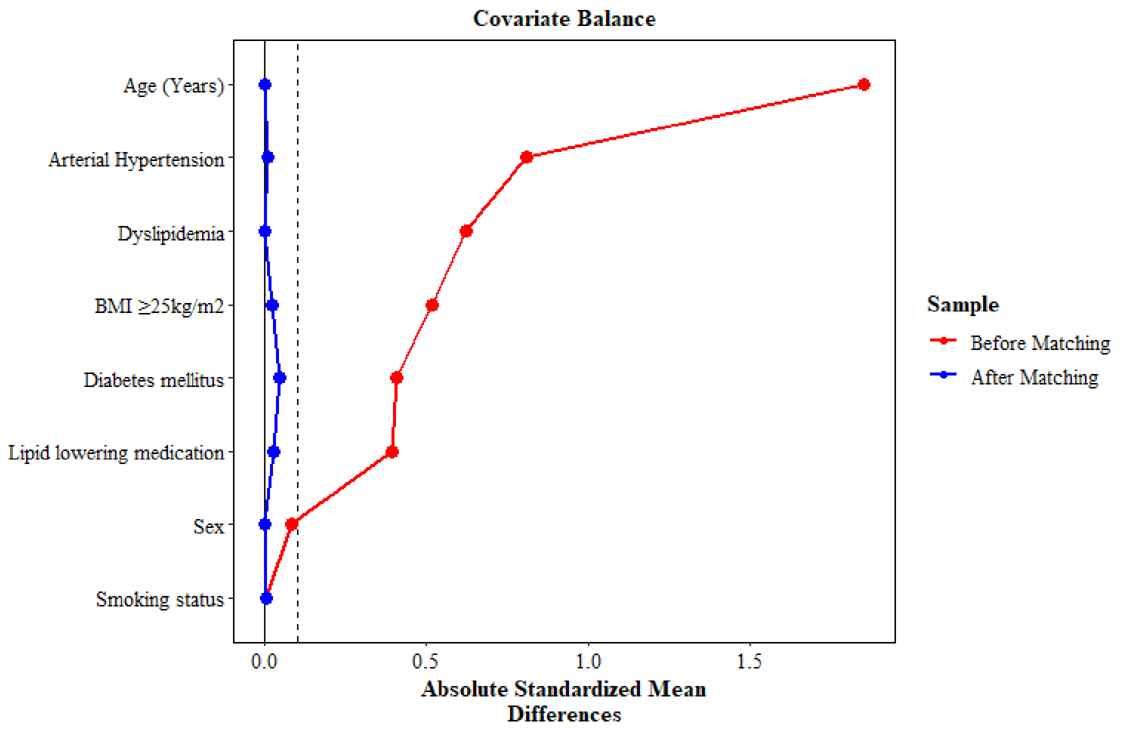
Covariate Balance
Age (Years)
Arterial Hypertension
Dyslipidemia
BMI ≥25kg/m2
Diabetes mellitus
Lipid lowering medication
Sex
Smoking status
0.0
0.5
1.0
1.5
Absolute Standardized Mean Differences
Sample
Before Matching
After Matching

**C. Venous thrombosis**

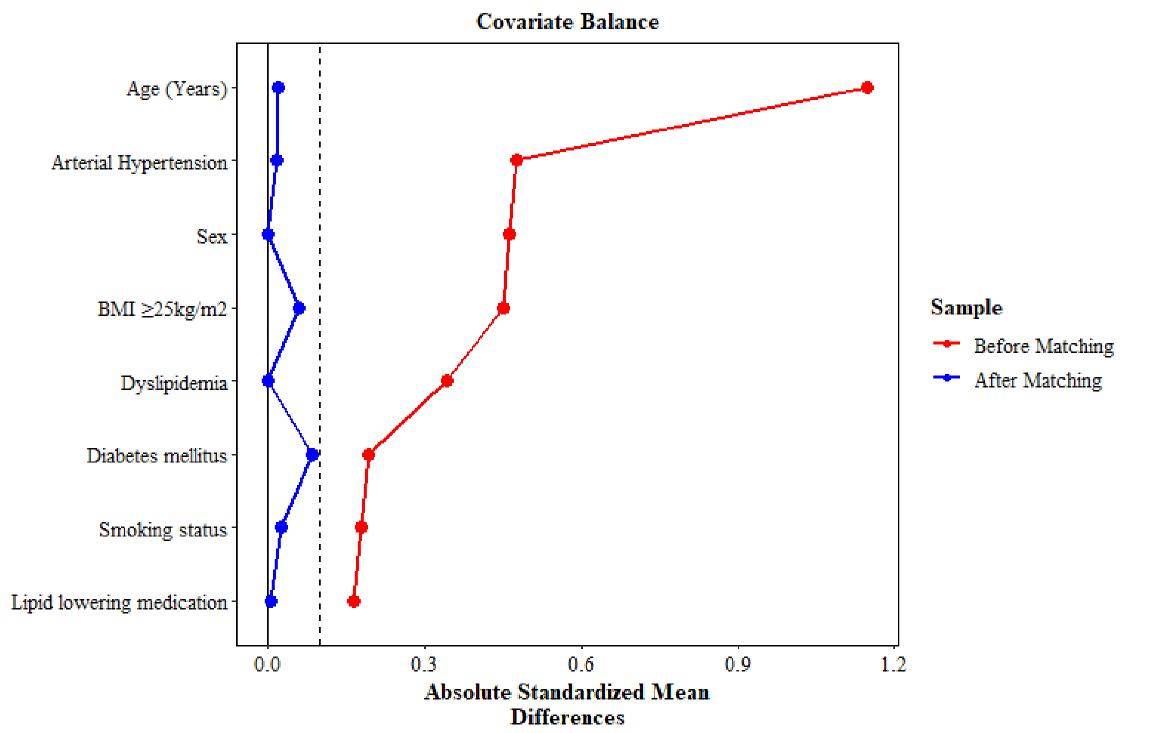


**Supplemental Figure 6.** Absolute standardized mean differences before and after propensity score matching.

Caption: Propensity score matching (PSM) was performed using logistic regression to estimate the probability of having a history of cardiovascular disease based on the following covariates: age, sex, body mass index (BMI) category, smoking status, arterial hypertension, diabetes mellitus, dyslipidemia, and use of lipid-lowering medication. Exact matching was applied on sex and dyslipidemia. Matching was conducted using nearest neighbor matching without replacement, with a 1:5 ratio (event to no event) and a caliper of 0.2.

**CVD:** The sample included 6,434 individuals before matching and 2,995 individuals after matching.

**ASCVD:** The sample included 6,434 individuals before matching and 2,460 individuals after matching.

**VT:** The sample included 6,434 individuals before matching and 1,466 individuals after matching.
